# SLEPR: A Sample-Level Enrichment-Based Pathway Ranking Method — Seeking Biological Themes through Pathway-Level Consistency

**DOI:** 10.1371/journal.pone.0003288

**Published:** 2008-09-26

**Authors:** Ming Yi, Robert M. Stephens

**Affiliations:** Advanced Biomedical Computing Center, Advanced Technology Program, SAIC-Frederick Inc., NCI-Frederick, Frederick, Maryland, United States of America; University of Michigan, United States of America

## Abstract

Analysis of microarray and other high throughput data often involves identification of genes consistently up or down-regulated across samples as the first step in extraction of biological meaning. This gene-level paradigm can be limited as a result of valid sample fluctuations and biological complexities. In this report, we describe a novel method, SLEPR, which eliminates this limitation by relying on pathway-level consistencies. Our method first selects the sample-level differentiated genes from each individual sample, capturing genes missed by other analysis methods, ascertains the enrichment levels of associated pathways from each of those lists, and then ranks annotated pathways based on the consistency of enrichment levels of individual samples from both sample classes. As a proof of concept, we have used this method to analyze three public microarray datasets with a direct comparison with the GSEA method, one of the most popular pathway-level analysis methods in the field. We found that our method was able to reproduce the earlier observations with significant improvements in depth of coverage for validated or expected biological themes, but also produced additional insights that make biological sense. This new method extends existing analyses approaches and facilitates integration of different types of HTP data.

## Introduction

Although the use of DNA microarrays and other high throughput (HTP) technologies is increasingly widespread and affordable, identifying the underlying biological themes from HTP data remains a major challenge in the arena of systems biology. Once data from these experiments has been normalized, a tremendous variety of software tools and methods are available for analysis of HTP data, primarily focusing on microarray data. These methods primarily rely on several categories of common gene-level approaches. One category of approaches is gathering patterned genes across samples or datasets by clustering (e.g., hierarchical [1], K-means [2], or SOM [3] methods, pattern extraction method [4]); Another category of common approaches is generating differentiated gene lists from two or more class contrasts using a variety of methods (Significance Analysis of Microarray [5], moderated t-test [6]–[7], LPE [8], FDR [9], as well as other gene selection methods including “unusual ratio method” [10], analysis of variance (ANOVA) related methods [11]–[16], Mixed Model Analysis [17]. A more recent approach, namely meta-analysis, looks for common signatures across multiple independent datasets by combining multiple statistical methods including simple t-test, FDR, and cross-validation into a single result [18].

In order to identify the biological themes embedded in such differentiated gene lists, the next step typically maps these genes to their pathways or networks. Further integration of this data with literature resources connects the identified genes with their potential biological roles [4], [19], www.ingenuity.com, www.genego.com. Alternatively, enrichment-based analysis [4], [20]–[22] can be applied to such a gene list to generate ranked functional categories (e.g., GO) or pathways based on their enrichment levels, so that the significantly enriched pathways and their associated genes can be easily identified as the primary biological themes. Additional efforts have been made using various algorithms and statistical methods for gene set-based group testing analysis [23]–[27]. Some very recent efforts explore the topology and architectures of the networks in conjunction with high-throughput data to seek biological scenarios [28]–[29].

The existence of high levels of natural variation within populations coupled with the observation that very slight changes in multiple relevant genes in a gene set can trigger biological changes has led to the development of several gene-set based or group testing methods: 1. over-representation analysis (ORA) [4], [20]–[22], [30]; 2. functional class scoring (FCS) [23]–[24], [27], [31]–[33]&semi; 3. global tests [34]; 4. module-level analysis scheme [35]–[37]; 5. singular value decomposition or SVD-based method [38]; 6. a network structure-based method [39].

Most, if not all of these methods directly use cross-sample evaluation for differentiated genes or ranking genes for further gene-set based methods, based on the assumption that, for a given phenotype (e.g. tumor vs. normal tissues, treated vs. control), any relevant genes should behave consistently across the samples or individuals within the studied population within their own class (e.g., Tumor or normal tissues). With existing group-test analysis methods such as GSEA [23]–[24], the assertion is made that in order to be significant, the data distribution per gene is significantly shifted between the contrasted classes. However, we challenge this notion with a new more general assertion that while biologically relevant genes may consistently behave in correlation with an associated phenotype across a population, it is even more likely that common pathways can be impacted through distinct gene events that are not reflected at the individual gene level across samples. Such pathway-level consistency seems particularly relevant considering the stochastic nature of many epigenetic events that lead to disease states. For example, a conventional t-test based approach is usually used to evaluate the statistical difference of genes among the individuals between two contrasted classes for a typical microarray dataset. For a specific gene, even with a statistically significant p-value, the expression of this gene in one or more samples of one class could have the same or even lower value than some samples from the other class, although the mean of expression for this gene in the first class is higher than that of the second one with statistical significance suggested by p-value. Thus, with the presence of such sample-level variants, this gene will still be considered as a differentiated gene and become included in the final differentiated gene list because it may have passed statistical criteria including p-value or FDR. This is a result of the fact that all variances of individual gene levels are aggregated into a single decision: differentiated gene or a single measurement: fold change or p-value. However, as we will discuss below, such sample-level variants or specificity in a class population, although some would be more common and some would be unique, can be both realistic and biologically relevant, since even for this same phenotype, other relevant genes could have changed or other types of changes for this gene, such as phosphorylation status or protein stability, which could not be captured by microarray, could happen in this individual. This may otherwise mask the real effect leading to the same or a similar phenotype. In short, multiple genes within a biological pathway could be impacted with the same net-effect on the pathway. Therefore what may be happening at a higher biological level (i.e., group of relevant genes, gene sets, pathways, functionally related genes) may have been excluded from the analysis if such sample-level specificity is not taken into account.

In order to capture the sample-level specificity of gene-level variance, we introduce a new concept - sample-level differentiated genes (SLDGs). These are defined as genes that are differentially expressed for a sample in one class when compared to the data distribution of the other class population. We believe that this new concept should accommodate both the gene-level changes identified by established methods but also sample-level specific changes related to the phenotype but only evident in individual samples. Although variations and even outliers that can be introduced by technical issues or experimental variations that have nothing to do with biological relevance, the chance that multiple related genes in a pathway or biological process have such issues simultaneously should be rare. Thus, SLDGs for each sample can be used as individual gene lists to evaluate the data consistency at the pathway-level using an ORA-like enrichment method. Instead of using summarized differentiated gene lists, SLDG lists are used to calculate enrichment levels of each term for each sample and a class-contrast based pathway-ranking method is then used to rank the most consistent and enriched pathways (or gene sets, GO terms). We named this method Sample-Level Enrichment-based Pathway-Ranking method (SLEPR).

In this report, we provide evidence that analyzing data at the level of functional categories, including well-defined or customized pathways and GO terms, for pathway-level consistency, may help understand the underlying biological themes at a higher level or in more detail than other methods provide. Furthermore, in addition to identifying potential biologically relevant processes or pathways, we also look for pathway-level differentiated genes from sample-level differentiated gene lists, which can be combined among sample populations to reveal a whole spectrum of genetic and biochemical changes associated with the phenotype in question. In contrast to conventional methods, we have extended the “differentiated” concept from the gene-level to the pathway-level, so that one can focus on a biological process or pathway that has consistent changes at the pathway-level rather than just individual gene-level across the samples or datasets in a study.

As a proof of concept, we have used this method to analyze several public microarray datasets with validated results and/or expected biological themes. We have found that the SLEPR method effectively reproduced the previously analyzed and experimentally validated results or generated analysis results that are consistent with biologically relevant expectations. In direct comparison with the GSEA method, we also found that the SLEPR method uncovered many other potentially biologically relevant pathways not identified by GSEA including many sample-specific genes that potentially cover the entire repertoire of candidate genes for pathways or gene sets that are associated with the expected phenotype. We hope that these results give a more complete picture of phenotype-wise genetic and biochemical changes and that this method will help derive biological themes from additional datasets measuring changes at different levels of regulation including transcription, protein expression, and phosphorylation when these data become increasingly available in the future.

## Results

### Overview: Sample-Level Enrichment-Based Pathway Ranking Method (SLEPR)

**Figure 1:**
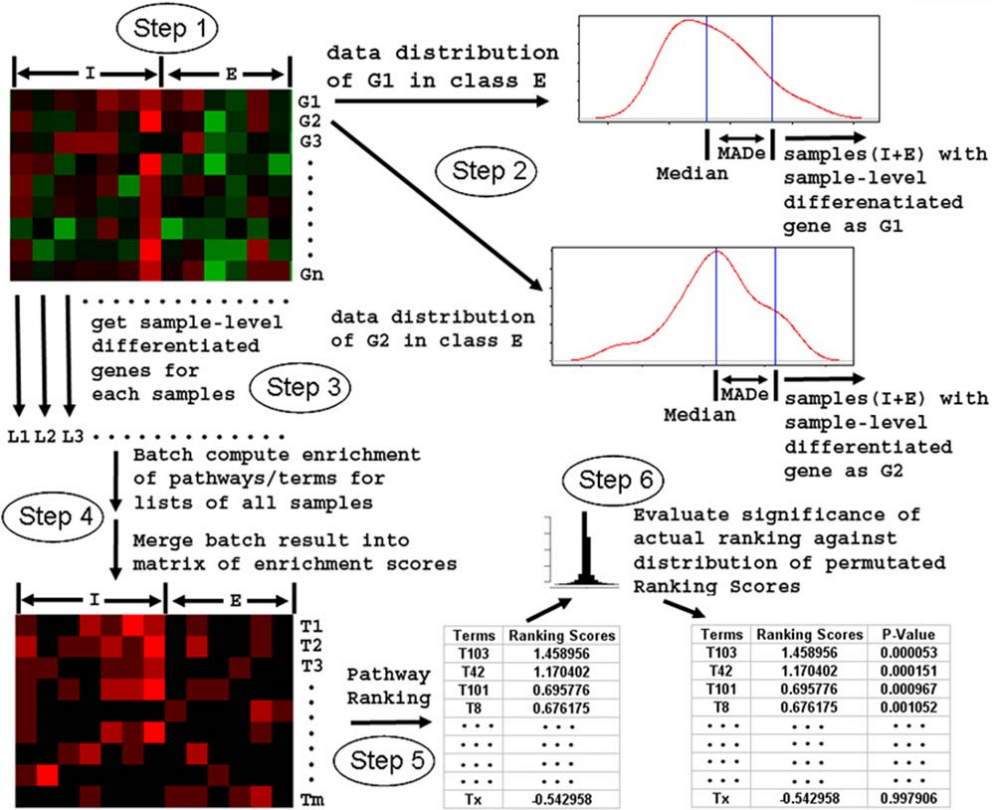
Schematic overview of SLEPR method (see Materials And Methods section for more details). The goal of SLEPR method is to use sample-level differentiated genes for each sample to capture the sample-level specificity for gene-level variance, and then use functional enrichment levels of these gene lists to evaluate pathway-level data consistency associated with the contrasted classes in study: Inclusion/Target class versus Exclusion/Background class (e.g., NGT versus DM2+IGT in the human type 2 diabetes mellitus (DM2) study [23]). Step 1 of SLEPR is to assign the samples to the Inclusion class (I) and Exclusion class (E). Then for each genes or features in study (i.e., G1, G2, G3…Gn), consider the data distribution and use median and MADe of data in samples of class E to set up the cutoff for sample-level differentiated genes for each genes (Step 2). Each gene Gi will have its own cutoff to determine if it is a sample-level differentiated gene. Gene Gi will be selected as the sample-level differentiated gene for a sample if the data of gene Gi in this sample is beyond the cutoff (Step 3). Each sample including samples from both I and E classes will have its own sample-level differentiated gene list (L1, L2, L3….) (Step 3). To determine the functional enrichment levels in any *a priori* defined gene sets, pathways, or functional categories (e.g., GO terms) for each of the sample-level differentiated lists, batch computation of Fisher's exact test based enrichment analysis is performed and the results are merged automatically into a matrix (e.g., Stanford format file) of enrichment scores which consists of enrichment scores of each sample from class I and E for each term (T1, T2, T3, …Tm), which are transformed as −log_10_(p-value) of Fisher's exact test p-values (Step 4). To determine whether a gene set, pathway, or functional category (e.g., GO term) is significant in terms of how consistent it is enriched across samples, a pathway ranking algorithm is applied to the enrichment score matrix to obtain pathway ranking scores, which considers both positive contribution of class I and negative contribution of class E from individual sample-level enrichment level (see details in Materials And Methods section) (Step 5). To determine the statistical significance of actual ranking of a gene set or a GO term in the contrasted classes: I versus E, the entire procedure (steps 1 to 5) is repeated 1000 times or more by simply permutating the class labels for each selected samples (Step 6). The pathway ranking scores of each term from each permutation are pooled together and used to build the empirically derived distribution of pathway ranking scores from the permutation procedure. The permutated p-value for each term is calculated as the fraction of random trials resulting in permutated pathway ranking scores higher than the actual score from the original sample assignments.

To overcome the issues and limitations of the gene-level consistency paradigm where data analysis primarily considers the change in gene behavior (e.g., expression) that consistently occurs in the majority of the sample population, we devised a simple approach, namely Sample-Level Enrichment-Based Pathway Ranking Method (SLEPR), which is schematically illustrated in Figure 1 and described in more detail in the methods section. One of the major goals of the SLEPR method is to consider sample-level specificity for gene-level variances, and place the identified genes in the context of *a priori* defined gene sets, annotated biological processes or pathways, functional categories (e.g., GO terms) and look for pathway-level consistency of enrichment effects from changes occurring at multiple related genes systematically. The goal is to accomplish this objective without sacrificing sensitivity in detecting those genes that do behave consistently within their class.

Using a recent diabetes study and their class contrast (e.g., highly expressed in individuals with NGT versus those with DM2: [23]), we first derived sample-level differentiated genes for each sample based on the data distribution of samples from the Exclusion/Background class using MADe-based statistics (Figure 1). The null hypothesis for SLEPR is that sample-level differentiated gene lists are random sets of genes with regard to their belonging to a particular sample group in a given class contrast. The alternative hypothesis is that sample-level differentiated genes are associated with the specific class assignment and therefore the biological phenotype under study. We also chose to allow the concept of the gene group to remain arbitrary and thus can include conventional gene sets, pathways, functional categories such as GO terms or simply genes with a particular transcription factor binding site and we will simply refer to these groups as gene sets henceforth. As a consequence, pathway-level consistent enrichment of genes annotated in a gene set within each of these sample-level differentiated gene lists would be biologically relevant to the phenotype being contrasted in the classes under study.

We then determine the functional enrichment levels in any *a priori* defined gene sets for each derived sample-level differentiated gene list. The derived enrichment scores are used to evaluate the pathway-level consistency of enrichment for gene sets using a pathway-ranking algorithm in the SLEPR method. The method considers both the positive contribution of class I (included) and the negative contribution of class E (excluded) to individual sample-level enrichment. This is done in order to reduce possible bias by using only samples from the Exclusion class as the reference background for cutoff determination for sample-level differentiated genes.

### Case Study 1: Human Diabetes Datasets

The human type 2 diabetes mellitus (DM2) dataset described previously [23] consists of 22,000 genes in skeletal muscle biopsy samples from 43 age-matched males: 17 with normal glucose tolerance (NGT), 9 with impaired glucose tolerance (IGT) and 17 with DM2. A goal of the original study [23] was to identify gene expression changes characteristic of DM2 and pre-defined gene sets for association with the disease phenotype. A novel method, called Gene Set Enrichment Analysis (GSEA), was developed to successfully identify as significant a set of genes involved in oxidative phosphorylation, although none of the individual genes had a significant difference in expression between the diagnostic categories [23]. Although GSEA successfully identified oxidative phosphorylation as one of major biological themes associated with the disease phenotype, subsequent studies revealed that the GSEA method was biased toward assigning higher enrichment scores to gene sets of large size [40].

In order to compare methods, we applied the SLEPR method to the same microarray dataset from the human type 2 diabetes mellitus study [23]. To ensure a fair comparison, we also implemented into the SLEPR method the same GSEA annotation database (also call MSigDB) [24] and compared the analysis results for the same database of gene sets.

For each single gene in our SLEPR method, we used the Exclusion/Background class as the background or reference distribution of data. In contrast to GSEA-based methods, instead of looking at the change in expression of genes between the two contrasted classes, our SLEPR method starts with sample-level differentiated genes by comparing data of each sample to the data distribution of samples of the Exclusion class. Thus, the expression of each gene from each individual sample (samples from both Inclusion and Exclusion classes) is compared to this background distribution of the same genes' expression and is defined as a sample-level differentiated gene for a sample if the expression of this gene in the corresponding sample has an expression level at a distance larger than MADe compared to the median of background data range for this gene from either one or both directions (see Materials And Methods section).

We used the NGT samples as the Inclusion class and IGT and DM2 samples together or DM2 samples only as our Exclusion class (see Materials And Methods section for class definition) and used GSEA annotations of gene sets or MSigDB (http://www.broad.mit.edu/gsea/) for SLEPR (Table 1, 2) and GSEA analysis (Table 3, 4). Our SLEPR method successfully identified 5 closely related terms: “mitochondrial genes” (two of them, annotated from different sources), “electron transport”, and “oxidative phosphorylation”, “PGC related genes” at the top of the ranked term list using either IGT+DM2 or only DM2 samples as Exclusion class (Table 1, 2), which is consistent with the previous analysis result of GSEA [23]. Particularly when both IGT and DM2 samples are used, these relevant terms appear to be even more significant and ranked as the top 5 terms (Table 1). However, the GSEA method only ranked 3 of these terms among the top list when using only DM2 samples as the Exclusion class (Table 4), and only one of these terms among the top list when using both IGT and DM2 samples as Exclusion class (Table 3). Thus, the SLEPR method appears to uncover more terms with expected biological relevance with higher significance scores than GSEA in both settings of class comparison, especially when both IGT and DM2 samples were pooled together as one class (Table 1, 3). The additional terms uncovered by SLEPR: “mitochondrial genes” and “PGC related genes”, which were absent from the GSEA results and have strong biological relevance with the previously identified and validated term “oxidative phosphorylation”: the mitochondria are well known as the cellular compartment where the oxidative phosphorylation reactions occur and PGC (or PGC-1)-responsive genes involved in oxidative-phosphorylation are coordinately downregulated in human diabetes [23]. The fact that these additional terms are closely related to electron transport and oxidative phosphorylation, suggests that SLEPR could potentially uncover more related biological themes, presumably due to our consideration of sample-level specificity for gene-level variances and pathway-level consistency across the population.

**Table 1:** Top ranked GSEA annotation terms in SLEPR Analysis Result for Comparison of NGT vs DM2+IGT in human DM2 data.

GSEA_TermName	GSEA_TermID	Combined Ranking	Permutated P_Val	FDR q_Val
Mitochondrial genes	HUMAN_MITODB_6_2002	1.009408	0.00011557	0.144
Mitochondrial genes	MITOCHONDRIA	0.96734041	0.00013403	0.0835
Genes involved in electron transport	ELECTRON_TRANSPORT_CHAIN	0.73313179	0.00033066	0.13733333
Oxidative Phosphorylation	MOOTHA_VOXPHOS	0.63908609	0.00048395	0.15075
PGC related genes	PGC	0.41673472	0.00146549	0.3652
RIBOSOMAL_PROTEINS	RIBOSOMAL_PROTEINS	0.26145395	0.00456661	0.9483

Microarray data for human type 2 diabetes mellitus (DM2) study [23] was re-analyzed with SLEPR method to compare either NGT versus IGT+DM2 (IGT+DM2 as Exclusion/Background class in SLEPR). One-side MADe method was used in SLEPR for selection of highly expressed genes as sample-level differentiated genes in the comparison of NGT versus IGT+DM2. Two-side MADe method was also used for selection of highly and lowly expressed genes as sample-level differentiated genes, and similar result was obtained (see Table S4). 1000 permutations were performed. Combined_Ranking: Combined ranking scores for terms; Permutated_P_Val: p-value of terms derived from permutated data; FDR_q_Val: FDR of terms derived from permutated data (see Materials And Methods section for details).

**Table 2 pone-0003288-t002:** Top ranked GSEA annotation terms in SLEPR Analysis Result for Comparison of NGT vs DM2 in human DM2 data.

GSEA_TermName	GSEA_TermID	Combined Ranking	Permutated P_Val	FDR_q_Val
Mitochondrial genes	HUMAN_MITODB_6_2002	2.33012855	0.0000025	0.003
Mitochondrial genes	MITOCHONDRIA	1.19377468	0.0000975	0.0585
PROPANOATE_METABOLISM	PROPANOATE_METABOLISM	0.87576134	0.00029917	0.11966667
PGC related genes	PGC	0.85709181	0.00031583	0.09475
Oxidative Phosphorylation	MOOTHA_VOXPHOS	0.83877765	0.0003425	0.0822
Genes involved in electron transport	ELECTRON_TRANSPORT_CHAIN	0.80602325	0.0003875	0.0775
RIBOSOMAL				
PROTEINS	RIBOSOMAL_PROTEINS	0.73004401	0.00051333	0.088
Downregulated in correlation with overt Alzheimer's Disease, in the CA1 region of the hippocampus	ALZHEIMERS_DISEASE_DN	0.43387443	0.00222333	0.3335

Microarray data for human type 2 diabetes mellitus (DM2) study [23] was re-analyzed with SLEPR method to compare either NGT versus DM2 (DM2 as Exclusion/Background class in SLEPR). One-side MADe method was used in SLEPR for selection of highly expressed genes as sample-level differentiated genes in the comparison of NGT versus DM2. 1000 permutations were performed. Combined_Ranking: Combined ranking scores for terms; Permutated_P_Val: p-value of terms derived from permutated data; FDR_q_Val: FDR of terms derived from permutated data (see Materials And Methods section for details).

**Table 3 pone-0003288-t003:** Top ranked GSEA annotation terms in GSEA Analysis Result for Comparison of NGT vs DM2+IGT in human DM2 data.

GSEA_TermName	GSEA_TermID	ES	NES	NOM p-val	FDR q-val	FWER p-val
Upregulated by expression of mutant MeCP2 (Rett syndrome) vs. wt MeCP2 in fibroblasts	RETT_UP	0.6723	1.830771	0.001**	0.93558	0.56
Granule constituents expressed during mouse promyelocytic cell line cell differentiation	LIAN_MYELOID_DIFF_GRANULE	0.5568	1.791872	0.002012	0.700521	0.683
Genes highly associated with medulloblastoma treatment failure	POMEROY_MD_TREATMENT GOOD_VS_POOR_DN	0.5402	1.720124	0.016327	0.971015	0.875
Regulated by UV-B light in normal human epidermal keratinocytes, cluster 8	UVB_NHEK3_C8	0.4442	1.685022	0.001**	1	0.931
Genes involved in electron transport	ELECTRON_TRANSPORT_CHAIN	0.3457	1.680579	0.060797	0.863601	0.937
Down-regulated in mycosis fungoides (cutaneous T-cell lymphoma) T-cells resistant to IFN-alpha, compared to sensitive parent cell line	IFNALPHA_RESIST_DN	0.5722	1.648226	0.014315	0.980393	0.968
Regulated by UV-B light in normal human epidermal keratinocytes, cluster 6	UVB_NHEK3_C6	0.4663	1.642358	0.018987	0.885222	0.979

Microarray data for human type 2 diabetes mellitus (DM2) study [23] was re-analyzed with GSEA method (using the newest version (v 2.0.1) GSEA tool [22]) to compare NGT versus IGT+DM2. 1000 permutations were performed. **: p-value(s) is adjusted to p = 1/number of permutation for p = 0 according to GSEA manual.

**Table 4:** Top ranked GSEA annotation terms in GSEA Analysis Result for Comparison of NGT vs DM2 in human DM2 data.

GSEA_TermName	GSEA_TermID	ES	NES	NOM p-val	FDR q-val	FWER p-val
Up-regulated following treatment with Et-743 at any timepoint in at least 8 of 11 sarcoma cell lines	ET743_SARCOMA_UP	0.4778	1.851262	0.0019455	0.835225	0.416
Oxidative Phosphorylation	MOOTHA_VOXPHOS	0.6187	1.844164	0.02	0.460014	0.446
Target genes down regulated by p53	KANNAN_P53_DN	0.6835	1.84351	0.005848	0.309037	0.447
Genes involved in electron transport	ELECTRON_TRANSPORT_CHAIN	0.594	1.840375	0.0217822	0.240362	0.46
p-regulated in liver, heart or kidney tissue from hypophysectomized rats (lacking growth hormone), compared to normal controls	HYPOPHYSECTOMY_RAT_UP	0.5129	1.776757	0.0040404	0.3918	0.664
Upregulated by expression of mutant MeCP2 (Rett syndrome) vs. wt MeCP2 in fibroblasts	RETT_UP	0.544	1.697153	0.0179641	0.740229	0.855
Genes down-regulated by LIF treatment (10 ng/ml, overnight) in AtT20 cells	ABBUD_LIF_DN	0.569	1.682703	0.0226804	0.727445	0.872
OXIDATIVE_PHOSPHORYLATION	OXIDATIVE_PHOSPHORYLATION	0.5167	1.653935	0.0459082	0.832665	0.908

Microarray data for human type 2 diabetes mellitus (DM2) study [23] was re-analyzed with GSEA method (using the newest version (v 2.0.1) GSEA tool [22]) to compare NGT versus DM2. 1000 permutations were performed. **: p-value(s) is adjusted to p = 1/number of permutation for p = 0 according to GSEA manual.

Interestingly, for many of the relevant terms identified by both SLEPR and GSEA, SLPER revealed a higher significance compared to GSEA in general. For example, although both SLEPR and GSEA detected “oxidative phosphorylation” as a top ranked term, the permutated p-values of “oxidative phosphorylation” derived from SLEPR (p = 0.00048 or p = 0.00034, Table 1 and 2) appear to be more significant than the GSEA method (p = 0.029 from original GSEA analysis [23], p = 0.02 with current version (v2.0.1) of GSEA) (Table 4) comparing NGT with only DM2 samples, and p = 0.3511 comparing NGT with both DM2 and IGT samples, which is not significant and ranked only at 165 (Table 3 and see the complete list of the result in Table S1). This observation is extended further by examining the FDR q-values of “oxidative phosphorylation”: FDR = 0.15 or FDR = 0.082 from SLEPR results (Table 1 and 2) compared to those from GSEA results: FDR = 0.446 and FDR = 1 (Table 4, S1).

In addition, the top ranked terms in the GSEA results appeared to have much higher FDR q-values (Table 3, 4) than those identified with SLEPR. Furthermore, in the SLEPR results, we observe a sharp rise in FDR q-values from 0.3652 to 0.9483 between terms No. 5 and 6 (Table 1) and from 0.088 to 0.3335 between terms No. 7 and 8 (Table 2), suggesting that these thresholds might be used to separate the identified terms into high and low priority groups.

**Figure 2:**
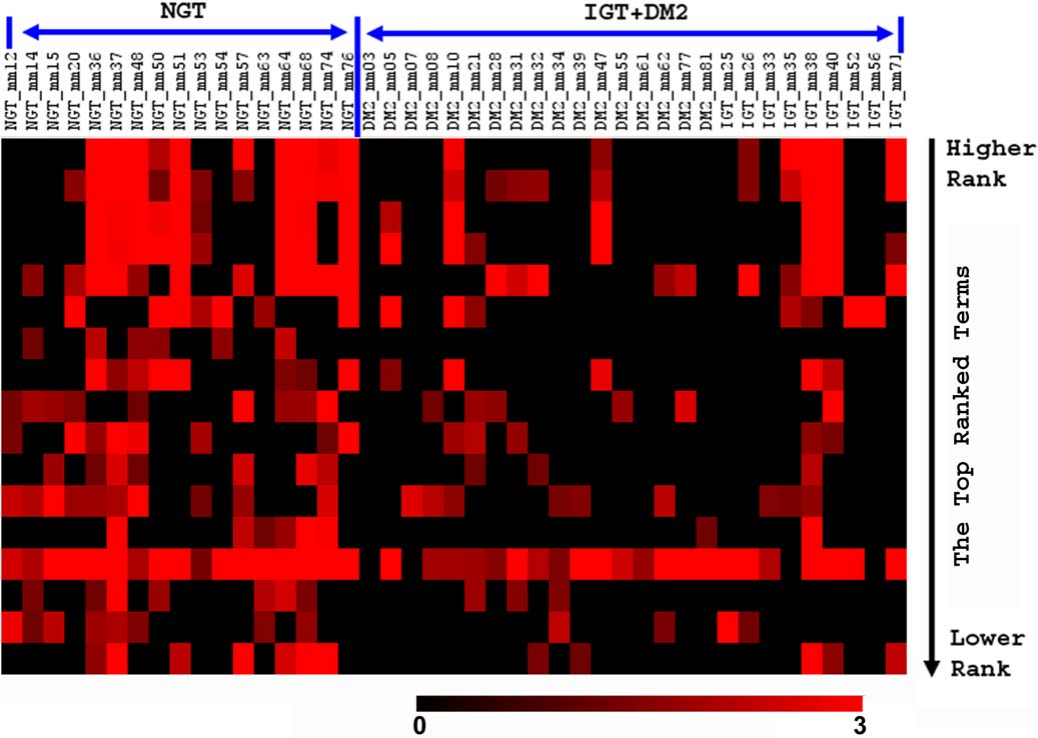
Heatmap of enrichment scores in all samples from NGT versus IGT and DM2 for the top 17 ranked terms of SLEPR result listed in Table S2. The enrichment scores, which in general derived from Fisher's exact test p-value using formula (−Log10(p-value)), were floored to 0 if the ListHits<2 or p-value>0.05. The rows of the heatmap are the ranked terms in the same order as in Table S2 (Top 7 of them shown in Table 1) from top to bottom with the higher ranks at the top. The gradient of red color in heatmap indicated the enrichment levels.

Since SLEPR is designed to uncover additional information from sample-level enriched genes, we wanted to evaluate how the top ranked terms are enriched in individual samples. To do this, we put the matrix of enrichment scores of 17 top ranked GSEA annotation terms of SLEPR result (Table 1, also see the complete list of the result in Table S2) into a heatmap for visualization (Figure 2). As expected, although the majority of NGT samples have enrichment scores at a significant level, a small portion of IGT or DM2 samples also have enrichment scores at significant levels for some terms, which expand to a larger portion of the samples for lower ranked terms that have relatively higher FDR q-values (Figure 2, Table S2).

In order to get a more complete picture of the genes that are involved in the top ranked terms found by the SLEPR method, we retrieved the pathway-level differentiated genes that are associated with one of the top ranked terms: “Oxidative Phosphorylation” from Table 1 using the newly developed WPS pipeline interface for pathway-level pattern extraction, described in a separate manuscript [4], Yi and Stephens, unpublished work. As expected, the pathway-level differentiated genes did not display an obvious gene-level consistency in their relative expression levels across the sample population even in the same class (Figure S1), although there may be higher levels expression in general in the Inclusion class (NGT) compared to Exclusion class (IGT+DM2). Interestingly, after ranking these genes with a method similar to a class difference ranking method used for term ranking (See Materials And Methods section), many of genes at the top of the ranked list showed strong relevance with diabetes, and metabolism (Table S3). Although the higher ranked genes appear to be distributed with higher frequency in sample population, each of the sample-level differentiated genes appeared widely varying among the samples. However, less variance was observed within the top ranked genes (Figure S2). This is consistent with the previous observation [23], and explains why common gene-level methods can not reveal the changes that occurred at pathway-level.

In order to determine how stable the SLEPR method is in terms of the choice of changed directions of sample-level differentiated genes and/or class setting, we also used SLEPR with a two-sided MADe option for highly or lowly expressed genes in NGT samples compared to DM2 samples for the same class setting (NGT versus DM2). Consistent with the one-sided MADe result (Table 1, 2), we found a very similar result with “Mitochondrial genes” (two of them from different resources: No. 1 and No. 5 terms), “PGC related genes” (No. 3 term), and “oxidative phosphorylation” as the top ranked terms in the lists with two-sided MADe options (highly expressed or lowly expressed in NGT) (Table S4). We also identified two or more of relevant terms including “Mitochondrial genes”, “PGC related genes”, “Oxidative Phosphorylation”, and “Genes involved in electron transport” as top ranked terms as well in many different class contrast settings: NGT versus DM2+IGT with two-sided MADe; DM2 lower than NGT with one-sided MADe; DM2+IGT lower than NGT with one-sided MADe; NGT versus IGT with one-sided MADe (data not shown).

Selection of cutoffs for significance testing in any gene-set analysis will have a dramatic impact on the number of pathways identified and the reliability of the results obtained. In order to determine the impact of the cutoffs for sample-level differentiated genes on the final SLEPR results, we used a series of different cutoffs for selection of sample-level differentiated genes for comparison of permutated p-values and ranks of SLEPR analysis results for human type 2 diabetes mellitus (DM2) data [23] with IGT and DM2 samples as the Exclusion/Background class and with one-side MADe method for highly expressed genes (as above). We selected the top 5 ranked terms from the original SLEPR result (Table 1) for comparison of permutated p-values and ranks for the same terms in SLEPR results with other cutoffs for selection of sample-level differentiated genes (Table 5). We found that, unlike the conventional ORA method [31], which usually uses a gene-level differentiated gene list, the SLEPR method appears to be quite resistant to the cutoff change effects. For a wide range of values for cutoffs of sample-level differentiated genes, SLEPR maintains the main biological themes in its analysis results in that all the top ranked terms based on the original 1× MADe cutoff are primarily ranked at the top level (most of the terms still remained at the top level: ranked within the top 10 functional terms and p-value less than or close to 0.01). This stability is an important property of the SLEPR method that would be beneficial, especially for the situation when different HTP samples with dramatically different dynamic ranges are being analyzed.

**Table 5:** Comparison of SLEPR results using a series of cutoffs for selection of sample-level differentiated genes.

TermName (TermID)	0.5XMADe		0.75XMADe		1XMADe		1.25XMADe		1.5XMADe	
	p-Value*	Rank	p-Value*	Rank	p-Value*	Rank	p-Value*	Rank	p-Value*	Rank
Mitochondrial genes (HUMAN_MITODB_6_2002)	0.150	153	3.08E-04	1	1.16E-04	1	6.58E-05	2	1.03E-05	1
Mitochondrial genes (MITOCHONDRIA)	0.170	179	6.38E-04	2	1.34E-04	2	2.22E-05	1	2.31E-05	2
Genes involved in electron transport (ELECTRON_TRANSPORT_CHAIN)	4.49E-03	7	8.82E-04	4	3.31E-04	3	1.75E-04	3	7.61E-05	4
Oxidative Phosphorylation (MOOTHA_VOXPHOS)	1.01E-02	9	6.91E-04	3	4.83E-04	4	2.22E-04	4	7.53E-05	3
PGC related genes (PGC)	1.05E-02	10	2.93E-03	6	1.47E-03	5	5.05E-04	6	6.21E-04	7

Comparison of permutated p-values and ranks of SLEPR analysis results of for human type 2 diabetes mellitus (DM2) data [23] with IGT and DM2 samples as Exclusion/Background class by using a series of different cutoffs (0.5, 0.75,1,1.25,1.5× of MADe) for selection of highly expressed genes as sample-level differentiated genes. The one-side MADe method selecting for highly expressed genes was used (see Materials And Methods section). The top 5 ranked terms from the case of 1XMADe are selected for comparison of permutated p-values and ranks for the same terms with those derived from other cutoffs. 1000 permutations were performed.

To compare how significant the enrichment score of a top ranked term from SLEPR result is on the DM2 dataset to that of a gene set by random chance, we generated 2000 randomly selected gene sets of the same size as the gene set “Oxidative Phosphorylation (MOOTHA_VOXPHOS)” (80 genes per gene set) from annotated genes in the GSEA database or MSigDB. Then the 80 genes of “Oxidative Phosphorylation” were mixed with the 2000 randomly selected gene sets to build up the synthetic database for SLEPR analysis using the same human DM2 dataset. As expected, “Oxidative Phosphorylation” was identified as the No. 1 top ranked term with FDR q-value as 0.016 when NGT versus IGT+DM2 class contrast was used, whereas the No. 2 ranked term (PermTerm157) has FDR q-value at as high as 0.502, indicating a “gap” between the real relevant terms and the accidental hit of a randomly selected gene set term (Table S5). A similar result was found using NGT versus DM2 contrast (data not shown).

### Case Study 2: GNF Human Tissue Datasets

We next used the SLEPR method to analyze another public microarray dataset: Affymetrix U133A tissue expression dataset, derived from 79 human tissues, from the Genomic Institute of the Novartis Research Foundation (GNF) and described previously [41]. In this case, we used both GSEA annotation and GO (gene ontology) biological processes as the input gene sets for SLEPR analysis. Specifically, we examined whether there were tissue-specific biological processes that are enriched in testis-related tissues that were highly ranked in SLEPR analysis when configured to select testis-related tissues as the Inclusion class and the other tissues as Exclusion class, using one-sided MADe method for highly expressed genes. The expected result was obtained in SLEPR analysis using either GSEA annotation or GO biological processes in WPS [4], Yi and Stephens, unpublished work, as shown in Table 6 for GSEA annotations and Table 7 for GO biological processes. There are two top ranked functional terms from GSEA annotations (the No. 1 and No. 2 ranked terms) that are directly related to testis-related gene expression as obviously described by the terms themselves (Table 6). As a comparison, the same data was analyzed using the GSEA method to compare the selected testis-related tissues with other tissues within the GNF dataset. As shown in Table 8, the top 2 GSEA terms obtained by GSEA method do not appear to be directly related to testis-related functions, whereas the same two terms that were found as the top 2 terms in the SLEPR method were only ranked as terms 3 and 4, respectively, in the GSEA analysis result. Interestingly, we also observed that there exists a sharp rise in FDR q-values from 0.121 to 0.417 in between No. 2 and 3 terms of GSEA result and in between No. 1 and No. 2 as well (Table 8), which seems to reduce the significance of No. 3 and 4 terms that do not appear to be directly relevant to the function of testis. This observation makes the GSEA result more difficult to interpret in that the testis-specific terms (No. 3 and 4 terms) appear to be less significant (Table 8). In contrast, we observed a sharp rise in FDR q-values from 0.0005 to 0.0437 between term No. 2 and 3 of the SLEPR result (Table 6) and such a “gap” in SLEPR result may help distinguish the testis-specific terms (The top 2 terms) from more generic terms (No. 3, and 4 term) (Table 6)

**Table 6:** Top ranked terms in SLEPR analysis result for testis-related tissues in GNF dataset.

GSEA_TermName	GSEA_TermID	Combined_Ranking	Permutated_P_Val	FDR_q_Val
Genes expressed specifically in human testis tissue	HUMAN_TISSUE_TESTIS	25.62201	7.29E-07	0.001
Testis related genes curated from the GNF normal tissue compendium	TESTIS_EXPRESSED_GENES	24.08417	7.29E-07	0.0005
Cell-cycle dependent genes regulated following exposure to serum in a variety of human fibroblast cell lines	SERUM_FIBROBLAST_CELLCYCLE	6.951284	9.56E-05	0.0437
50 top ranked SAM-defined over-expressed genes in each subgroup__PR	ZHAN_MM_CD138_PR_VS_REST	6.523997	0.000106	0.0365

Microarray data for GNF human tissues study [41] was analyzed with SLEPR method to compare testis related tissues (total 5 testis related tissues selected: Testis Germ Cells, Testis Interstitial, Testis Leydig Cell, Testis Seminiferous Tubule, Testis) (as Inclusion/Target tissues in SLEPR) to the other tissues (total 74 tissues) (as Exclusion/Background class in SLEPR) as for GSEA annotated gene sets. In SLEPR method, one-side MADe method for selection of highly expressed genes as sample-level differentiated genes was used. 1000 permutations were performed.

**Table 7:** Top ranked GO Biological Processes terms in SLEPR analysis result for testis-related tissues in GNF dataset.

TermName	Term	Combined_Ranking	Permutated_P_Val	FDR_q_Val
spermatogenesis	GO∶0007283	17.21279	6.71E-07	0.0005
male gamete generation	GO∶0048232	17.21279	6.71E-07	0.0005
sexual reproduction	GO∶0019953	16.27175	6.71E-07	0.000333
reproduction	GO∶0000003	16.17275	6.71E-07	0.00025
gametogenesis	GO∶0007276	13.95838	6.71E-07	0.0002
nuclear division	GO∶0000280	4.461007	0.000178	0.044167
M phase	GO∶0000279	4.361697	0.000195	0.041429

Microarray data for GNF human tissues study [41] was analyzed with SLEPR method to compare testis related tissues (total 5 testis related tissues selected: Testis Germ Cells, Testis Interstitial, Testis Leydig Cell, Testis Seminiferous Tubule, Testis) as Inclusion/Target tissue to the rest of the tissues (74 other tissues) as Exclusion/Background class for testis-specific GO biological processes, using one-side MADe method for selection of highly expressed genes as sample-level differentiated genes. 1000 permutations were performed.

**Table 8:** Top ranked terms in GSEA analysis result for testis-related tissues in GNF dataset.

GSEA_TermName	GSEA_TermID	ES	NES	NOM p-val	FDR q-val	FWER p-val
50 most interesting genes upregulated by the combination of TSA and DAC in at least one of four pancreatic cancer cell lines, but not in normal (HPDE) cells	TSADAC_PANC50_UP	0.571787	2.3071	0.001**	0.002	0.004
Up-regulated 2 hours after VEGF treatment in human umbilical vein endothelial cells	VEGF_HUVEC_2HRS_UP	0.562051	2.01	0.001**	0.077	0.121
Testis related genes curated from the GNF normal tissue compendium	TESTIS_EXPRESSED_GENES	0.868249	1.8032	0.001**	0.271	0.417
Genes expressed specifically in human testis tissue	HUMAN_TISSUE_TESTIS	0.904183	1.7737	0.001**	0.255	0.474

Microarray data for GNF human tissues study [41] was analyzed with GSEA method [23] (Using the newest version (v2.0.1) GSEA tool [24]) to compare testis related tissues (total 5 testis related tissues selected: Testis Germ Cells, Testis Interstitial, Testis Leydig Cell, Testis Seminiferous Tubule, Testis) to the other tissues (total 74 tissues) as for GSEA annotated gene sets. 1000 permutations were performed. **: p-value(s) is adjusted to p = 1/number of permutation for p = 0 according to GSEA manual.

**Figure 3:**
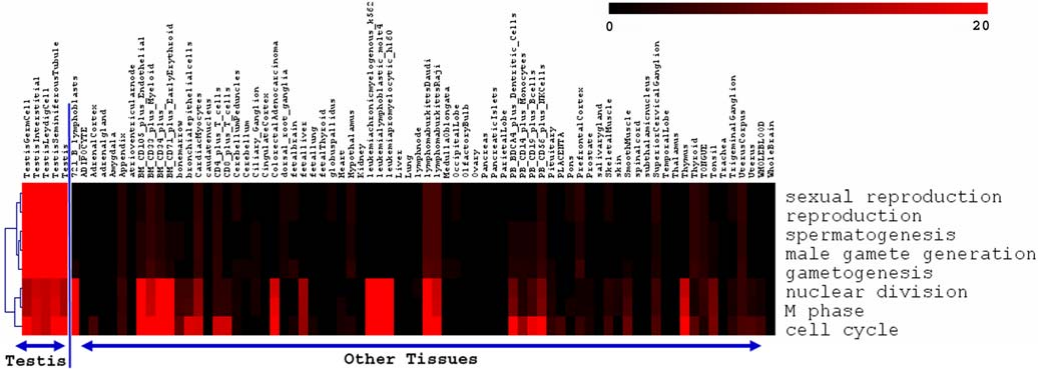
Heatmap of enrichment scores of sample-level differentiated genes of all samples in human GNF tissue dataset [41] for the top 8 ranked GO biological process terms shown in Table 7. The enrichment scores, which in general derived from Fisher's exact test p-value using formula (−Log10(p-value)), were floored to 0 if the ListHits<2 or p-value>0.05. The rows of the heatmap are the terms and columns are tissue samples from the dataset. The gradient of red color in heatmap indicated the enrichment levels.

Similarly, most of the top ranked terms in the SLEPR result using GO biological processes are related to testis-specific functions such as spermatogenesis, and male gamete generation (Table 7). Interestingly, we again observed a sharp rise in FDR q-values from 0.0002 to 0.044167 in between No. 5 term (gametogenesis) and No. 6 term (nuclear division). Interestingly, all of the top 5 terms are testis-specific terms whereas beginning at No. 6 term, the terms that ranked below are more generic terms, which suggested a useful FDR “gap” between significant and insignificant terms that seems to reflect the difference in biological relevance or specificity. This observation is also evident in a heatmap of enrichment scores in that these testis-specific functional terms (The top 5 terms in Table 7) are enriched consistently and more specifically in testis-related tissues compared to rest of the tissues in the dataset (Figure 3).

We also asked the same question as to muscle-related tissues in the data. In contrast to testis, where essentially a single biological process is performed, we felt that muscle would represent a more diverse tissue type, since it is involved in more processes. We expected that muscle-specific functions or processes would be highly ranked in SLEPR analysis if we chose to select muscle-related tissues as the Inclusion class and the other tissues as Exclusion class, and used a one-sided MADe method to include highly expressed genes as the sample-level differentiated genes. As expected, we found that the two top ranked terms are muscle-specific functions in the SLEPR analysis result using GO biological processes (Table 9). Despite the fact that the muscle-related tissues we selected are quite divergent in that they include cardiac myocytes, smooth muscle, heart, and skeletal muscle, which were selected together as Inclusion class, the divergence among these tissues might be quite small compared to the divergence among other tissues, which makes the pathway ranking still favor muscle-specific terms. This shows another strength of the SLEPR method in that it may still be able to catch the pathway-level difference when there is divergence or large variation amongst the samples in the same phenotypic class. This is useful in that in cases where sufficient samples are not available from an individual study, they may be able to be combined with samples from other studies.

**Table 9:** Top ranked GO Biological Processes terms of SLEPR result for muscle-related tissues in GNF dataset.

TermName	Term	Combined_Ranking	Permutated_P_Val	FDR_q_Val
muscle contraction	GO∶0006936	5.035783	0.000195	0.289
muscle development	GO∶0007517	3.766843	0.000438	0.325
cell-cell signaling	GO∶0007267	3.115529	0.000755	0.373667
morphogenesis	GO∶0009653	2.51698	0.001281	0.47525
development	GO∶0007275	2.509388	0.001292	0.3836
organogenesis	GO∶0009887	2.434454	0.001375	0.34
cell motility	GO∶0006928	2.410239	0.00141	0.298857
striated muscle contraction	GO∶0006941	1.781977	0.002482	0.4605
regulation of body fluids	GO∶0050878	1.602872	0.003043	0.501778
angiogenesis	GO∶0001525	1.40132	0.003982	0.5909

Microarray data for GNF human tissues study [41] was analyzed with SLEPR method to compare muscle related tissues (total 4 muscle related tissues selected: Cardiac Myocytes, Heart, Skeletal Muscle, Smooth Muscle) as Inclusion/Target tissue to the rest of tissues (74 other tissues) as Exclusion/Background class for testis-specific GO biological processes, using one-side MADe method for selection of highly expressed genes as sample-level differentiated genes (see Materials And Methods section). 1000 permutations were performed.

Since both testis-related tissues and muscle-related tissues have a limited number of samples: 5 and 4, respectively, which are only a small portion of total 79 tissues in the dataset, there is a possibility that the small number of the samples in the Inclusion class may cause a bias favorable to the inclusion class. To test this possibility, we chose a related tissue type with more samples in the dataset to test whether the sample-size influences the SLEPR method. We choose neural or brain-related tissues as our Inclusion class with a total sample size of 24 and other tissues as the Exclusion tissues. We ran the SLEPR analysis on GO biological processes and used the one-sided MADe method to include highly expressed genes as the sample-level differentiated genes. Interestingly, the sample size of the Inclusion class does not seem to influence the SLEPR result - the top 4 ranked GO terms are indeed very specific to neural-related biological processes (Table S6).

As described above, among the several tissue types (e.g., testis, muscle, neural-related tissues) we have chosen from this human tissue dataset to test the SLEPR method, every time it successfully identified many top-ranked terms that are directly relevant to what is expected from the tissue types that were chosen. In order to further characterize the pathway-level differentiated genes at the top of these ranked term lists, we retrieved the associated genes for the top ranked testis-specific terms (Table 6) or muscle-specific terms (Table 9) using the new features in the pathway pattern extraction pipeline of the WPS program [4], Yi and Stephens unpublished work. Interestingly, we found that many of pathway-level differentiated genes associated with these testis-specific or muscle-specific terms are expressed at relatively higher level in only a small portion of these selected testis-related or muscle-related tissues although some genes are highly expressed in other tissue types. Figure S3 shows the expression patterns of such genes, which are associated with the top 2 ranked muscle-specific terms from SLEPR analysis shown in Table 9 (i.e., muscle contraction and muscle development). This observation suggests that individual sample-level variations widely exist across the sample population, even for the genes that are involved in these highly relevant biological processes. This suggests that the commonly used gene-level analysis methods may fail to identify such relevant pathway-level differentiated genes.

### Case Study 3: Prostate Cancer Datasets

We also used the SLEPR method to analyze a well studied prostate cancer microarray dataset: Affymetrix U95a dataset, derived from 25 human prostate cancer tissues and 9 nonmalignant tissues described previously [42], [43]. In this case, we used both GSEA annotation and KEGG pathway collections [4]. Interestingly, in our SLEPR analysis for both highly and lowly expressed genes (two-sided MADe option for SLEPR) (Table 10), we found that the top ranked functional gene sets from GSEA annotation are obviously cancer-related including: 1) “Sixty-seven genes commonly upregulated in cancer relative to normal tissue from a meta-analysis of the OncoMine gene expression database” (p = 5.43E-7, FDR q-value = 0.00011) and 2) “Genes highly expressed in hepatocellular carcinoma with poor survival” (p = 5.43E-7, FDR q-value = 2.94E-5). Another top ranked gene set: “Genes up-regulated by MYC in P493-6 (B-cell)” (p = 5.43E-7, FDR q-value = 3.45E-5), may be also consistent with the previous observation as to the presence of varying amount of B-cells within these tumors [42] and the fact that c-MYC is a proto-oncogene that is commonly activated in a variety of human tumors and has been shown to promote tumor angiogenesis [44]. In contrast, in the GSEA analysis result (Table 11), only two of the top terms were found in the top list: “Genes up-regulated by MYC in P493-6 (B-cell)” (p = 0 (actually p = 0.001 based on 1000 permutation according to GSEA manual), FDR q-value = 0.031) and “Sixty-seven genes commonly upregulated in cancer relative to normal tissue from a meta-analysis of the OncoMine gene expression database” (p = 0.004, FDR q-value = 0.058). However, for the third term: Genes highly expressed in hepatocellular carcinoma with poor survival, GSEA only ranked it at position 124 in the list with a very low significance level (p = 0.167, FDR q-value = 0.467) (Table S7).

**Table 10:** Top ranked terms in SLEPR analysis results for a well-studied prostate cancer dataset.

GSEA_TermName	GSEA_TermID	Combined_Ranking	Permutated_P_Val	FDR q_Val
Sixty-seven genes commonly upregulated in cancer relative to normal tissue, from a meta-analysis of the OncoMine gene expression database	CANCER_NEOPLASTIC_META_UP	7.312481	5.43E-07	0.000111
Genes downregulated in response to glutamine starvation	PENG_GLUTAMINE_DN	6.578343	5.43E-07	6.25E-05
These are genes identified by simple statistical criteria as differing in their mRNA expresssion between WTs and fetal kidneys LOW	LI_FETAL_VS_WT_KIDNEY_UP	5.961163	5.43E-07	0.00004
Genes 2fold upregulated by insulin	ROME_INSULIN_2F_UP	5.905375	5.43E-07	3.7E-05
Genes up-regulated by MYC in P493-6 (B-cell)	SCHUMACHER_MYC_UP	5.762658	5.43E-07	3.45E-05
Genes highly expressed in hepatocellular carcinoma with poor survival.	HCC_SURVIVAL_GOOD_VS_POOR_DN	5.449725	5.43E-07	2.94E-05

A well studied prostate cancer dataset [42], [43] was analyzed with SLEPR method. SLEPR method used the 25 tumor samples as Inclusion/Target class compared to 9 nonmalignant tissues as Exclusion/Background class for GSEA annotated terms, by two-sided MADe method for selection of both highly and lowly expressed genes as sample-level differentiated genes. Only top ranked functional terms were shown in the table (the chromosomal location-based annotation terms were taken out for simplicity). 1000 permutations were performed.

**Table 11:** Top ranked terms in GSEA analysis results for a well-studied prostate cancer dataset.

GSEA_TermName	GSEA_TermID	ES	NES	NOM p-val	FDR q-val	FWER p-val
Genes up-regulated by MYC in P493-6 (B-cell)	SCHUMACHER_MYC_UP	0.6769	2.043	0.001*	0.031466	0.039
Genes overexpressed in polyclonal plasmablastic cells (PPCs), mature plasma cells isolated from tonsils (TPCs), and mature plasma cells isolated from bone marrow (BMPCs), as compared to B cells purified from peripheral blood (PBBs) and tonsils (TBCs)	TARTE_PC	0.6101	1.954	0.0019193	0.07472	0.144
Genes downregulated in response to glutamine starvation	PENG_GLUTAMINE_DN	0.4842	1.939	0.001**	0.058919	0.167
Genes downregulated in response to leucine starvation	PENG_LEUCINE_DN	0.5067	1.911	0.001**	0.066378	0.238
Downregulated in HL-60 promyeloid leukemic cells after treatment with the cytotoxic drug cantharidin	CANTHARIDIN_DN	0.6218	1.903	0.001*	0.059042	0.26
Genes downregulated in response to rapamycin starvation	PENG_RAPAMYCIN_DN	0.5000	1.891	0.0020576	0.060076	0.309
Sixty-seven genes commonly upregulated in cancer relative to normal tissue, from a meta-analysis of the OncoMine gene expression database	CANCER_NEOPLASTIC_META_UP	0.6634	1.883	0.0042105	0.058265	0.335

A well studied prostate cancer dataset [42], [43] was analyzed with GSEA method (Using the newest version (v2.0.1) GSEA tool [24]). GSEA compared the tumor samples to the nonmalignant tissues. Only top ranked functional terms were shown in the table (the chromosomal location-based annotation terms were taken out for simplicity, the full ranked list can be obtained from table S7). 1000 permutations were performed. **: p-value(s) is adjusted to p = 1/number of permutation for p = 0 according to GSEA manual.

We also ran SLEPR analysis using KEGG annotation for this dataset with both highly and lowly expressed genes (two-sided MADe option for SLEPR). Surprisingly, we saw a pathway “Cholera – Infection” at the top of the ranked list (Table S8). As found by others using the same dataset with the conventional ORA method [43], “Cholera – Infection” is related to tumorgenesis since this pathway contains genes such as adenylate cyclase signaling and phospholipase C that are changed in tumor cells. In addition, “Integrin-mediated cell adhesion” was found as the next top ranked pathway (Table S8), which has been suggested as a target pathway in many other studies to achieve an optimization of anticancer treatments, probably through interfering with anti-apoptotic signaling [45] and/or metastasis. However, this pathway was not found by conventional ORA analysis or gene-level based analysis [42], [43], probably due to the dispersion of the data or a lack of consistency at the gene-level for genes in this pathway.

## Discussion

More and more evidences have shown that conventional gene-level analysis methods seeking biomarkers or differential genes encountered limitations and difficulties from both statistical and biological sides [46]–[47]. As with the analysis approaches discussed in the Introduction section, most, if not all of these gene selection methods consider the global behavior of individual genes across the sample population in one class compared to another class as the basis for grouping by applying various statistics including fold change, p-value and FDR. The genes associated with the phenotype of interest that behave more consistently within both sides of the contrasted classes will be favorably selected as differential genes and pursued in follow-up studies. The assumption that the most critically involved genes tend to behave in a similar way between samples within each class is well founded in many cases, especially for single genes that cause rare diseases. However, the inherent complexity of biological systems, the multiple stages where protein function can be regulated, and the high overall levels of individual variations, suggest that this approach may miss important aspects of biology.

Interestingly, Chinnaiyan's group has hypothesized that many oncogenes may exhibit marked over-expression only in a subset of tumor samples and traditional analysis methods such as t-statistic has limitation to detect them [48]. Consequently, they proposed a novel method, commonly known as “Cancer outlier profile analysis” or COPA, which can effectively uncover such oncogene outlier expression profile [48]. Such efforts have been improved and extended by a few other groups [49]–[52]. These methods are great renovations over the conventional t-statistic based or other gene-level consistency-based methods. However, since these methods mainly focus on extremely expressed genes or outliers and still consider all of their statistics at gene-level, although they may use other genes' data to estimate the significance of their statistics, they are still considered as gene-level approaches.

In the current study, we explored a new method termed SLEPR, which considers the possibility that genetic impacts leading to class distinctions can occur, and consequently be measured, at the pathway level rather than at the individual gene level. Our method is motivated not only similarly as COPA methods intended for oncogene outliers occurred in only a subset of tumor samples, but also more importantly by the observation that many diseases are not simply caused by single genes, including complex diseases such as cancers, heart diseases, and hypertension, which have been shown to be caused by mutations in multiple genes in the same or related pathways or caused by single but different genes in individuals that causing biological changes in the same or related pathways among the population [53]–[56]. This is what we proposed as pathway-level consistency instead of gene-level consistency that traditional methods are based on.

In order to evaluate inter-sample consistency at the pathway-level, we introduce a new concept: sample-level differentiated genes (SLDGs). Unlike conventional approaches for gene-level differentiated genes, which use data from the sample population of one class compared to those of the other class, the SLDGs are based on the data of each individual sample from both sides of the contrasted class compared to the Exclusion/Background class. We make no assumptions of the data distribution and use MADe, a factored Median Absolute Deviation (MAD), which has more robust statistics compared to standard deviation-based statistics and is largely unaffected by the presence of extreme values [57]–[58] (see Materials And Methods section). Since we select SLDGs from the higher and/or lower ends (distance of MADe from the median) of that genes' data distribution in the Exclusion/Background class sample, the method should capture both gene-level and pathway-level differential effects. It should be noticed that our SLDGs are different from the outlier genes selected by COPA methods mentioned early in that SLDGs are sample-wise genes called on behalf of each sample, whereas outlier genes from COPA methods are called population-wise in spite of considering the occurrence of outlier genes in only a subset of population. The second difference is that the SLDGs not only include outliers as conventionally defined, but also cover high and/or low end of expressers as the basis for the next-step analysis of SLEPR.

It is possible that when SLEPR method selects SLDGs with 1XMADe as the cutoff, the chance of outlier data leading to mistakenly selecting genes would be higher. The reason that we did not filter out the outlier data in advance is that we believe some outliers have biological relevance, just like what the COPA methods seek for, which is not caused by experimental issues. Simply filtering these genes out before evaluating their biological relevance could lead to loss of important information. Since we use the Fisher's exact test based enrichment method for pathway-level consistency analysis, the outliers could work together with other relevant genes of high and/or low end expressers to contribute to enrichment scores, which otherwise would be reduced to less significant levels.

We have tested our method with a series of constants (0.5, 0.75, 1.25, 1.5; Table 5; even 1.75, data not shown) that multiply MADe as the final cutoffs for selecting sample-level differentiated genes rather than the default setting of 1.0 and our MADe selections are quite stable in the final pathway-ranking results in that they pick up the same set of terms as highly or top ranked terms. This suggests that the SLEPR method is quite stable in terms of the cutoff for selection of genes, which is in contrast to the conventional ORA method using a single summary gene list as a starting point that has been claimed to be sensitive to the cutoff used for getting the gene lists [31].

Thus, sample-level differentiated genes represent a robust input starting point for subsequent SLEPR enrichment analysis using Fisher's exact test as the basis for discrimination of changed pathways. The derived enrichment score for each sample from both sides of the contrasted classes was used to evaluate the pathway-level consistency. We further evaluated the consistency across samples between both Inclusion and Exclusion classes for the best pathway that was present at a higher frequency and higher enrichment magnitudes in samples of the Inclusion/Target class but not in Exclusion/Background class, or with less frequency or lower enrichment magnitudes. This is the basic rationale that we used to set up the pathway-ranking algorithm in SLEPR method.

As we have shown in example datasets, the SLEPR method worked quite well and was able to not only reproduce the previously analyzed and experimentally validated results (the human DM2 dataset, Table 1,2; prostate cancer dataset, Table 10) or generated analysis results that are consistent with biologically relevant expectations (the GNF tissue dataset, Table 6, 7 and 9; prostate cancer dataset, Table 10), but also may have provided more opportunity to study those highly ranking terms using the pathway-level differentiated genes derived from the corresponding sample-level differentiated genes. We also suggest that one could even rank these genes for their possible relevance to a phenotype of interest between the contrasted classes as we showed for our pathway-ranking algorithms (Figure S1, Table S3, also see Materials And Methods section).

As a comparison with other group test methods such as the GSEA method [23], we carefully selected three datasets, which are well characterized public datasets that either have had the results validated (the human DM2 dataset, prostate cancer dataset [23], [42]) or have clear biological expectations based on the nature of the studies they were derived from (the GNF human tissue dataset [41]). Thus, the analysis results from these datasets can be easily interpretated and compared for different analysis methods. In addition, we are confident the choice of these well-characterized data should be better than any simulated data or synthetic datasets, since they carry natural noise levels from both experimental and biological variations. In fact, we did a couple of tests using synthetic databases derived from randomly selected genes forming artificial gene sets mixed with a real gene set and SLEPR worked very well in these cases (Table S5).

We have found that in all the head-to-head comparisons with the GSEA method, our SLEPR method consistently did a better job or got at least compatible results with those from the GSEA method (Table 1, 2 vs. 3, 4; 6 vs. 8; 10 vs.11). First of all, our method was more sensitive than the GSEA method at uncovering biological themes with higher significance in general. Such a conclusion was drawn by comparing p-values, FDR q-values and rankings obtained by both methods for the sense of relative significance considering all the terms in the results, taking into account the difficulty in directly comparing the p-values and FDR q-values derived from the two quite different methods with differences in algorithms and rationales. For example, the highly ranked term “oxidative phosphorylation” has higher significance revealed by SLEPR than by GSEA as mentioned in the Result section (Table 1–4). Secondly, as evident in Table 1 to [Table pone-0003288-t002] [Table pone-0003288-t003] 4 and 10 to11, our SLEPR method was more powerful than the GSEA method in terms of finding more relevant terms with a broader scope for biological relevance functionally linked to the phenotypes under study. For example, 5 related terms were consistently uncovered by SLEPR (Table 1, 2) compared to only 1 or 3 of them that were found by GSEA with the same class setting, respectively (Table 3, 4). Thirdly, our method was consistent and powerful in the analysis result with flexible inclusion of relatively diversified but related samples into analysis, especially when the intermediate class samples were included. For example, as evident in Table 1 to [Table pone-0003288-t002] [Table pone-0003288-t003] 4, particularly in Table 1 vs. 3, where DM2 and IGT were pooled together as one class in class comparison with NGT (NGT vs. DM2+IGT) based on the observation that the intermediate class IGT is more similar to DM2 in phenotype than NGT, SLEPR has consistently uncovered many of the relevant functional terms similar to class comparison of NGT vs. DM2 (Table 1, 2), whereas GSEA failed to do so (Table 3, 4). In addition, SLEPR was able to find consensus of underlying functions within related but different samples, which can be only “loosely” defined as one class (e.g., testis-related tissue in Table 6 and 8; muscle-related tissues in Table 9; neural-related tissue in Table S6). This would give SLEPR more flexibility and power to overcome the variations and naturally existing noise in biology samples to find the major biological consensus. Fourthly, in a number of cases mentioned in the RESULTS section, the appearance of FDR “gap” (the sharp rise) in SLEPR results implicated a potential statistical threshold that may have biological relevance, which may help users distinguish the significant terms from non-significant ones and easily draw a line to select the terms for further investigation. Fifthly, in contrast to the potential bias in GSEA method that higher enrichment scores were assigned preferentially to gene sets of large size [40], no such bias exists in SLEPR method (Figure S4). Lastly, since our SLEPR method begins with sample-level differentiated genes in contrast to the gene-level consistency-based gene ranking in the GSEA method, SLEPR is designed to capture the sample-wise gene-level changes taking into account individual variations and specificity over the population and obviously would cover more possible gene-level changes in the population with the phenotype of interest. This is another benefit that SLEPR is designed to pursue. Consequently, SLEPR would be able to retrieve more possible relevant genes as pathway-level differentiated genes that may account for phenotype of interest, which GSEA or other group test methods may have missed due to the fact that they only consider the genes with better across-sample data behavior (e.g., SNR on top of mean and standard deviation of both contrasted classes in GSEA [32]; correlated expression pattern [33]; fold change or ratio in between two classes [27]).

It is very important to emphasize that SLEPR is neither a simple extension of GSEA or other group test methods with similar analysis goals nor COPA methods specifically looking for outlier genes, but rather a novel pathway analysis method in terms of its unique concepts and methodology. Unlike others, SLEPR does not attempt to rank the genes or derive conventional differentiated genes at the beginning; instead, it just collects the potential genes for each sample that behave unusually compared to the population. Then SLEPR ranks the pathways or terms based on how consistent the enrichment levels of the terms amongst the selected sample-level differentiated genes of each sample. It is SLEPR that points out the new concept for the analysis: pathway-level consistency as the basis for analysis, which is not considered in any of the other analysis methods including GSEA.

In conclusion, the SLEPR method represents a novel way to analyze high throughput data through pathway-level consistencies that have been proven to be effective in uncovering biological themes. Since sample-level differentiated genes can be selected from datasets measuring changes at different levels of regulation, including transcription, protein expression, and phosphorylation occurring in the same individual samples, all HTP data measuring these changes in the systems biology era can be integrated and included in SLEPR method. Furthermore, we feel that using sample-level rather than gene-level enrichment as a starting point may represent a much more robust and versatile approach for integration of data from multiple sources as new technologies advance the ability to assess regulatory networks at multiple levels.

## Materials and Methods

Three public microarray datasets [23], [41], [42] were obtained from the original publications and used for purpose of demonstration for SLEPR method. All the described procedures for the SLEPR method are implemented into and as a part of newly developed pathway pattern extraction pipeline in the original WPS program developed previously [4], which can be downloaded from the WPS website (http://www.abcc.ncifcrf.gov/wps/wps_index.php). The details and program interface of the pathway pattern extraction pipeline in WPS program are described elsewhere in a separate manuscript (Yi and Stephens unpublished work). The following sections are details of those procedures, which were also schematically illustrated in Figure 1.

### Inclusion/Target Class versus Exclusion/Background Class

For a given dataset intended for contrast studies (e.g., NGT vs. IGT+DM2; tumor vs. normal tissues; muscle tissues vs. other tissues), we separated all of the samples from the dataset into two classes representative of interested contrast: Exclusion/Background class *E* (e.g., normal tissues) as the class for background of data measurement and the interested class: Inclusion/Target class *I* (e.g., tumor tissues) as the target sample group to make comparison with Exclusion/Background class. *E* and *I* is exchangeable and also do not have to cover all the samples in datasets, dependent upon the questions to address. The same sample can not be selected into *E* and *I* classes at the same time. Therefore, if *T_I_* as total number of samples from Inclusion class *I* and *T_E_* as number of samples from Exclusion class *E*, and there are total T samples in dataset. Thus, we have *T_I_*+*T_E_*< = T.

### Select Sample-Level Differentiated Genes for Each Sample in Dataset

For each single gene *k* in a dataset, its data in sample *j* is denoted as D_k,j_ (*k*: gene *k*; *j*: sample *j* in the dataset, either in Class I or Class E). To decide whether gene *k* is a sample-level differentiated gene for sample *j*, we first used data of gene *k* in samples within Exclusion/Background class *E* as background distribution of data to create a cutoff threshold C*_k_* for gene *k*, where we have *C_k_* = *MADe*({D*_k_*_,*j*_}), where j = 1,2,… *T_E_*; *T_E_* is number of samples in Class *E* in the dataset.

Function *MADe*() is defined as following: for n values *x_i_* (i = 1,2,..n) in a data set *X*:
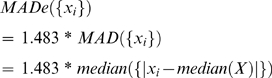
where the inner median *median*(*X*) is the median of the set *X* and the outer median is the median of the n absolute values of the deviations about the inner median. MAD is conventional Median Absolute Deviation and 1.483 is the scaling factor, which make *MADe* comparable with a SD (Standard Deviation), although *MADe* is more robust than SD and unaffected by the presence of extreme values or outliers [57]–[58]. We also define median expression level *M_k_* for gene *k* for samples in Exclusion class E as:
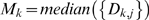
where j = 1,2,… *T_E_*; *T_E_* is number of samples in class E in the dataset.

There are two MADe-based methods for selecting sample-level differentiated genes: one-sided MADe method and two-sided MADe method. The one-sided MADe method intends to only select sample-level differentiated genes from one direction of changes (e.g., only up-regulated genes or only down-regulated genes) in each sample. In contract, the two-sided MADe method intends to select sample-level differentiated genes from both directions of changes (e.g., pooled highly expressed genes and lowly expressed genes together) in each sample.

For one-sided MADe method, there are two options for selecting genes: higher side (right-side) or lower side (left side) sorting options, which will select only highly expressed (e.g., up-regulated) genes, or only lowly expressed (e.g. down-regulated) genes as sample-level differentiated genes compared to the data distribution of Exclusion/Background class, respectively. For higher side sorting option, considering gene *k*, if and only if *D_k_*_,*m*_> = *M_k_*+*C_k_*, gene *k* will be selected as a sample-level differentiated genes (e.g., highly expressed) for sample m. For lower side sorting option, considering gene *k*, if and only if *D_k_*_,*m*_< = *M_k_*−*C_k_*, gene *k* will be selected as a sample-level differentiated gene (e.g., lowly expressed) for sample m; where sample m is one of the selected samples in the dataset (either a sample from Class *I* with *T_I_* samples or Class *E* with *T_E_* samples).

For two-sided MADe method, considering gene *k*, either if *D_k_*_,*m*_> = *M_k_*+*C_k_* or if *D_k_*_,*m*_< = *M_k_*−*C_k_*, gene *k* will be selected as sample-level differentiated gene (e.g., highly expressed or lowly expressed ) for sample m, where sample m is one of the selected samples in the dataset (either a sample from Class *I* with *T_I_* samples or Class *E* with *T_E_* samples). This method will pool together the genes that have changed at either direction (e.g., highly or lowly expressed) into sample-level differentiated lists.

Each time sample-level differentiated genes are selected, an intermediate result file can be created, in which a similar binary data matrix with value of either 1 or 0 was generated like the original data matrix, except that for each data point in the original matrix, say, a data point for gene g and sample s, the data was transformed to either 1 if gene g was selected as sample-level differentiated genes for sample g, or 0 if not. The interface for creating such an intermediate result file is implemented into WPS [4] and described in a separate manuscript (Yi and Stephens unpublished work). Such an intermediate result file is useful if one wants to further pursue the importance of each of the pathway-level differentiated genes involving in the studied phenotype with class-ranking method described as below.

### Compute for Enrichment Scores for Each Sample-Level Differentiated Genes into an Enrichment Score Matrix

Once the sample-level differentiated gene lists are sorted into individual files in a file folder or directory using WPS program interfaces and utilities of pathway pattern extraction pipeline described in a separate manuscript (Yi and Stephens unpublished work), enrichment scores can be computed in a batch mode for each of these gene lists and merged into a stanford format file. Briefly, Fisher's exact test is performed based on 2×2 contingency tables (whether a gene is in the given list or not vs. whether this gene is associated with a pathway/term or not, described previously [4] for each term for each list). All Fisher's exact test results are ranked based on the p-values for each list and stored as individual files for further merging. During the merging process, all the p-values are transformed by a formula (−Log_10_(p-value)) into enrichment scores, where appropriate filtering is applied, typically, ListHits<2 or p-value>0.05 will be used to floor the enrichment scores to 0; otherwise, −Log_10_(p-value) will be the enrichment scores. The data matrix of enrichment scores without any flooring or filtering (i.e., original enrichment scores (−Log_10_(p-value))) may be also obtained from a program interface described in a separate manuscript (Yi and Stephens unpublished work).

### Pathway-Ranking for Enrichment Score Matrix

For each term in the enrichment score matrix, let the enrichment score of sample *i* in pathway or term t as ES*_i,t_*, *T_I_* as total number of samples from Inclusion class *I* and *T_E_* as number of samples from Exclusion class *E*.

In order to get the Pathway-ranking score for a given pathway or term t, we need compute for two intermediate ranking scores for each term t: class difference ranking scores and p-value sum ranking scores. Class difference ranking scores reflect the difference in the percentages of samples in class *I* and class *E* with significant enrichment scores. p-value sum ranking scores reflect the difference in the magnitude of the enrichment scores in samples of class *I* and samples of class *E*.

*Cfc* is defined as the Cutoff for class difference ranking score, as default, *Cfc* = −log_10_(0.05) = 1.3 (one can change the default in program interface to other desired value). First, we compute for class difference ranking score CDR for pathway or term *t*, which is defined as *CDR_t_*, we have:
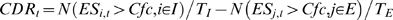
Where *N*(*ES_i_*_,*t*_>*Cfc*, *i*∈*I*) refers to the number of samples in class I with enrichment score larger than the *Cfc*; *and N*(*ES_j_*_,*t*_>*Cfc*, *j*∈*E*) refers to the number of samples in class E with enrichment score larger than the *Cfc*.

Then, we compute for p-value sum ranking scores for pathway or term *t*, which is defined as *PSR_t_*, we have:
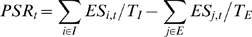
Where 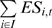 refers to the sum of enrichment scores for term *t* of all samples in class *I* and 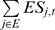 refers to the sum of enrichment scores for term *t* of all samples in class *E*. Thus, to compute the pathway-ranking score for a given pathway or term *t*, which referred as *PR_t_*, we have:

If both *CDR_t_* and *PSR_t_* are less than 0, *PR_t_* is computed as *PR_t_* = (−1)*CDR_t_*PSR_t_; otherwise, *PR_t_* is computed as: *PR_t_* = CDR_t_*PSR_t_.

### Estimation of Significance of Ranked Pathways/Terms

The statistical significance of a given pathway ranking score *PR_t_* for a given pathway or term *t* is assessed with permutated p-value using permutation testing of class assignments of each sample from both Inclusion and Exclusion classes (e.g., whether a sample has a phenotype of DM2 versus NGT in the human type 2 diabetes mellitus (DM2) dataset [23]). Briefly, we permutate the sample labels among total selected samples including both class I and class E. For each permutation, we re-calculate pathway-ranking score for each term *t* as permutated *PR_t_*. This procedure was repeated 1000 (default setting) or more times. The permutated p-value for each term *t* is calculated as the fraction of random trials resulting in permutated pathway-ranking scores no less than *PR_t_*. The FDR q value is also calculated based on this null distribution derived from permutation as followed: to compute an FDR q value, for a given pathway-ranking score *PR_t_*, the FDR is the ratio of the percentage of all pathway-ranking scores derived from the permutated data, which are no less than *PR_t_*, divided by the percentage of observed pathway-ranking scores derived from the original data, which are no less than *PR_t_*. Because such permutation tests randomize the class assignments of samples from both sides of contrasted classes, it is a test of the dependence of the actual class assignment for each individual sample, which is characteristic of the phenotype under study.

### Retrieval and Ranking of the Associated Genes for Significant Ranked Terms as Pathway-Level Differentiated Genes

The ranked pathways with significant permutated p-values, FDR, and/or rankings may be used for retrieval of their associated genes from the sample-level differentiated genes in samples of Inclusion class. The retrieval can be easily done using newly developed WPS pipeline interface for pathway-level pattern extraction, described in a separate manuscript (Yi and Stephens unpublished work). The retrieved genes can be ranked in a way similar to the class ranking method used for pathway ranking, using the subset of data for these genes derived from the intermediate result file created when selecting the sample-level differentiated genes as described above.

Within the retrieved associated genes, we compute for class difference ranking score CDRG for gene *g*, which is defined as *CDRG_g_*, and each transformed value TV (i.e. 1 or 0) in the data matrix of an intermediate result file referred as *TV_s_*_,*g*_ for sample s and gene g, then we have:
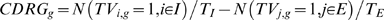
Where *N*(*TV_i_*_,*g*_ = 1, *i*∈*I*) refers to the number of samples in class I with transformed value in the intermediate file equal to 1; *and N*(*TV_j_*_,*g*_ = 1, *j*∈*E*) refers to the number of samples in class E with transformed value in the intermediate file equal to 1.

We define these genes with CDRG larger than 0 as pathway-level differentiated genes, which may potentially represent the whole repertoire of alternations occurring at gene level within the engaged pathway in association with the class contrast or compared phenotypes. Such ranking of pathway-level differentiated genes can be used for evaluating the significance or the probability of the involvement of these genes related to the interested phenotype in the dataset under study.

## Supporting Information

Figure S1 Sample-wise gene-level variations shown by the heatmap of z-scores for pathway-level differentiated genes, which are associated with one of the top terms (Oxidative Phosphorylation) from the SLEPR result in Table 1. The z-scores of these genes were computed using all samples from both Inclusion and Exclusion classes, and were displayed in the heatmap using color gradient for their values as red for positive z-scores and green for negative z-scores, black for scores of 0. The z-scores are calculated on each gene basis. For each sample, the z-score (also referred as standard score sometime) of an intended gene is derived by subtracting the population mean of this gene from the original data of the corresponding sample of this gene and then dividing the difference by the population standard deviation of this gene. In general, a positive z-score indicates a relatively higher expression level of a gene in the corresponding sample over the sample population for this gene; negative for a lower expression; 0 for average expression.
(1.41 MB TIF)

Figure S2 Heatmap of call values for sample-level differentiated genes, which are associated with one of the top terms (Oxidative Phosphorylation) from the SLEPR result in Table 1. The call value is 1 if the gene is called as sample-level differentiated genes for the corresponding sample, 0 if not. The genes were ranked in a way as described in Materials And Methods Section, and were displayed in the heatmap in the order of ranks from top to bottom with higher ranked genes at the top.
(1.17 MB TIF)

Figure S3 Sample-wise gene-level variations shown by the heatmap of Z-scores for sample-level differentiated genes, which are associated with the No. 1 (Muscle contraction) and No. 2 (Muscle development) terms from the SLEPR result in Table 9. The z-scores of these associated genes were computed using all samples from both Inclusion and Exclusion classes, and were displayed in the heatmap using color gradient for their values as red for positive z-scores and green for negative z-scores, black for scores of 0.
(1.37 MB TIF)

Figure S4 No bias for the SLEPR ranking scores vs. sizes of gene sets. A pdf file with several plots showing no bias for the SLEPR ranking scores vs. sizes of the gene sets: for distribution of SLEPR pathway ranking scores vs. gene set sizes (with different gene set size windows) and histogram distribution of gene set sizes for data in Table S2, Table S4, and Table S6 (for all terms or different numbers of top ranked terms in the tables).
(1.92 MB PDF)

Table S1 The complete list of result of GSEA analysis result for NGT vs. IGT+DM2 comparison, which has the top ranked terms shown in Table 3
(0.19 MB XLS)

Table S2 The complete list of result of SLEPR analysis result of GSEA annotations for NGT vs. IGT+DM2 comparison with one-side MADe option for highly expressed genes in NGT, which has the top ranked terms shown in Table 1.
(0.33 MB XLS)

Table S3 The complete list of ranked pathway-level differentiated genes of the No. 1 term (Oxidative Phosphorylation) in Table 1. The call values for sample-level differentiated genes of these genes are also included in the file.
(0.06 MB XLS)

Table S4 The complete list of SLEPR analysis result of GSEA annotations for NGT vs. DM2 comparison with two-side MADe option for highly or lowly expressed genes in NGT.
(0.30 MB XLS)

Table S5 The complete list of SLEPR analysis result of a synthetic database that consisted of 2000 randomly selected gene sets (with matched size of gene set) mixed with the known gene set of “Oxidative Phosphorylation” from MSigDB. NGT versus IGT+DM2 class contrast was used with one-side MADe option for highly expressed genes in NGT.
(0.22 MB XLS)

Table S6 The complete list of SLEPR analysis result of GO biological processes for comparison of neural or brain-related tissues vs. other tissues with one-side MADe option for highly expressed genes in neural or brain-related tissues.
(0.28 MB XLS)

Table S7 The complete list of result of GSEA analysis result for tumor vs. normal comparison, which has the top ranked terms shown in Table 11
(0.16 MB XLS)

Table S8 The complete list of SLEPR analysis result of KEGG pathways for comparison of tumor vs. normal with two-side MADe option for highly or lowly expressed genes in tumors.
(0.02 MB XLS)
