# Supplementary figures and images for: SLEPR: A Sample-Level Enrichment-Based Pathway Ranking Method — Seeking Biological Themes through Pathway-Level Consistency
Source: PLoS One. 2008 Sep 26;3(9):e3288. doi: 10.1371/journal.pone.0003288 (PMC2546449; doi:10.1371/journal.pone.0003288)

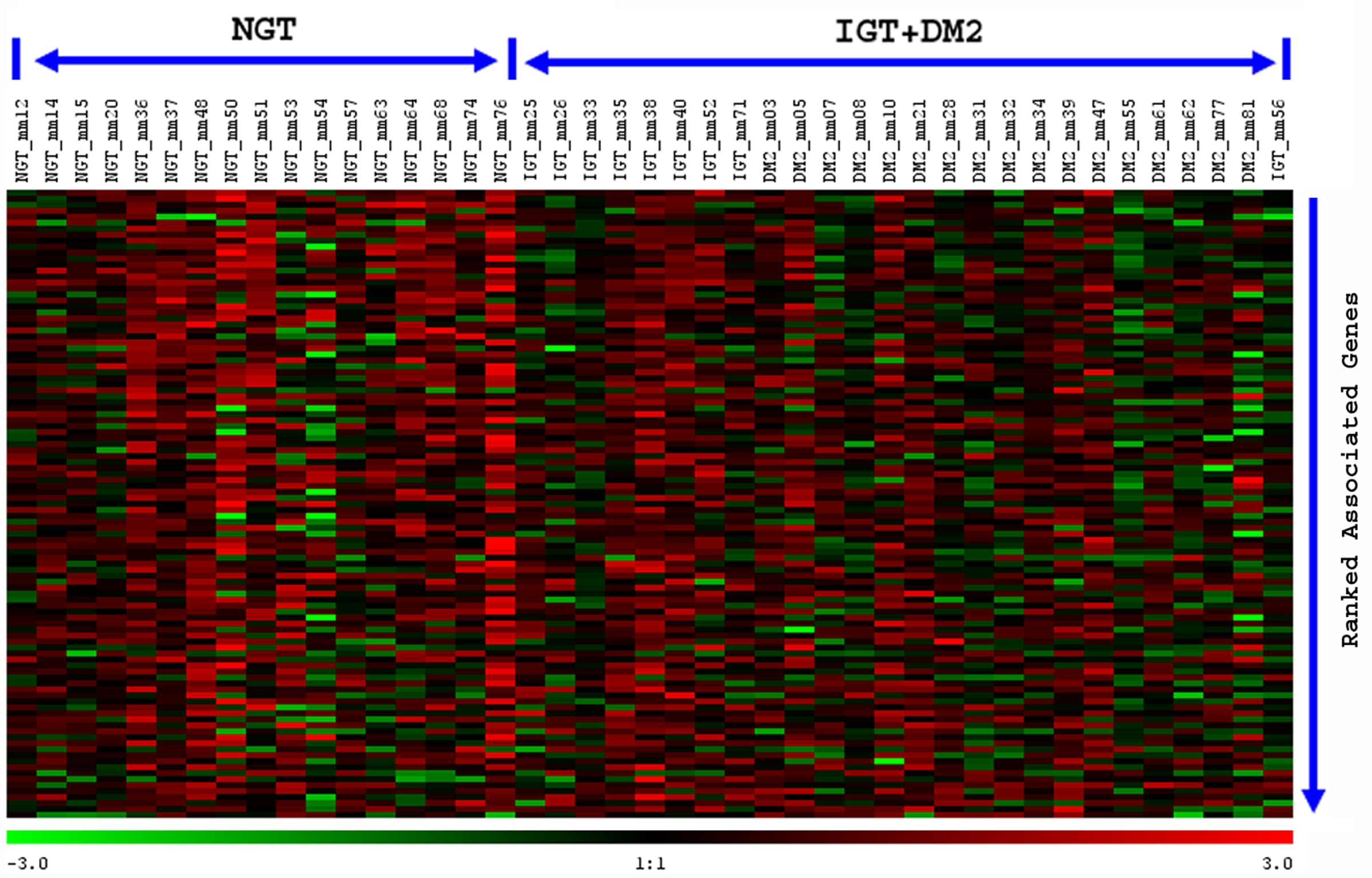

Supplement: Figure S1 — Sample-wise gene-level variations shown by the heatmap of z-scores for pathway-level differentiated genes, which are associated with one of the top terms (Oxidative Phosphorylation) from the SLEPR result in Table 1. The z-scores of these genes were computed using all samples from both Inclusion and Exclusion classes, and were displayed in the heatmap using color gradient for their values as red for positive z-scores and green for negative z-scores, black for scores of 0. The z-scores are calculated on each gene basis. For each sample, the z-score (also referred as standard score sometime) of an intended gene is derived by subtracting the population mean of this gene from the original data of the corresponding sample of this gene and then dividing the difference by the population standard deviation of this gene. In general, a positive z-score indicates a relatively higher expression level of a gene in the corresponding sample over the sample population for this gene; negative for a lower expression; 0 for average expression. (1.41 MB TIF) [file pone.0003288.s001.tif]

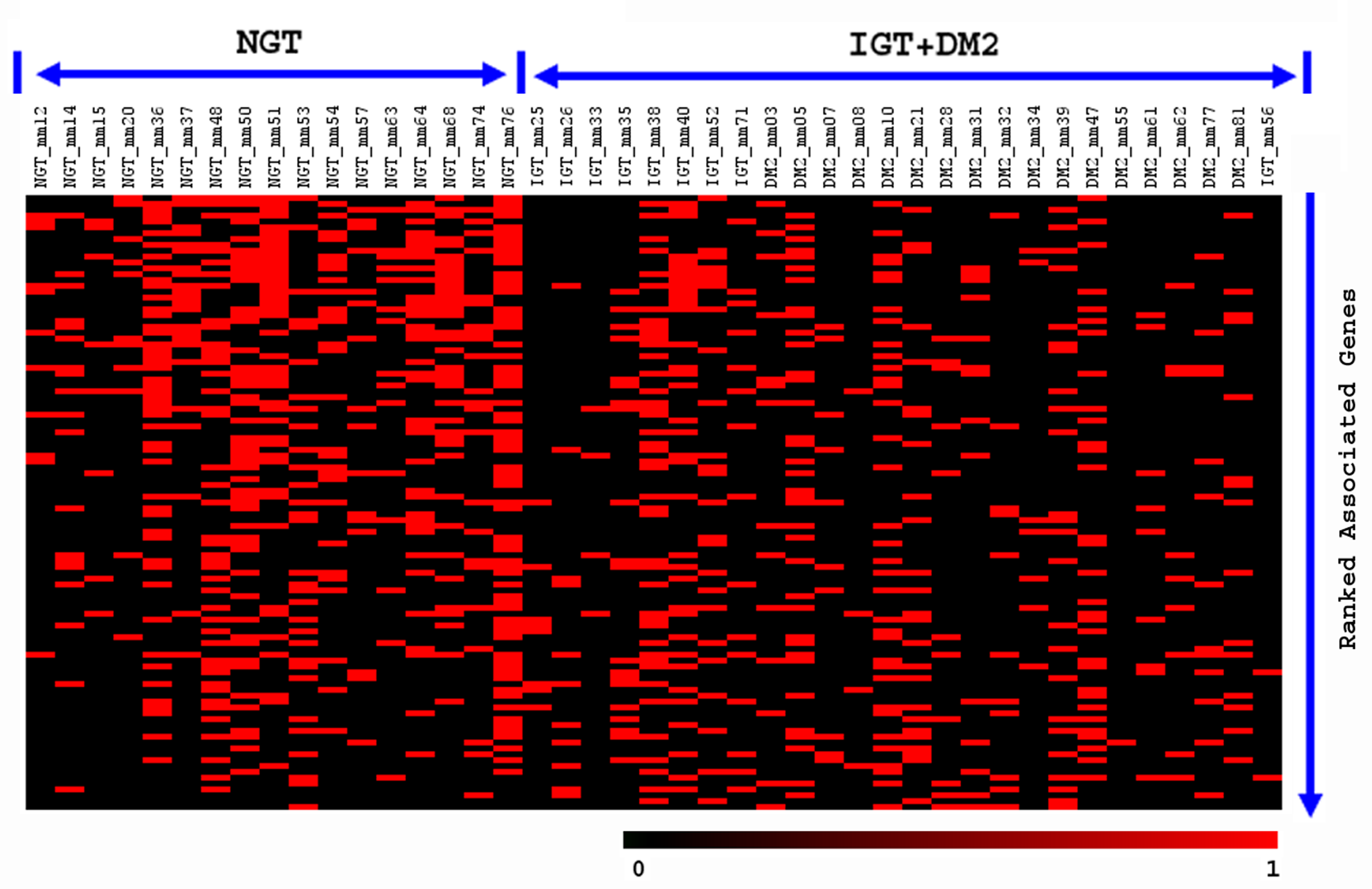

Supplement: Figure S2 — Heatmap of call values for sample-level differentiated genes, which are associated with one of the top terms (Oxidative Phosphorylation) from the SLEPR result in Table 1. The call value is 1 if the gene is called as sample-level differentiated genes for the corresponding sample, 0 if not. The genes were ranked in a way as described in Materials And Methods Section, and were displayed in the heatmap in the order of ranks from top to bottom with higher ranked genes at the top. (1.17 MB TIF) [file pone.0003288.s002.tif]

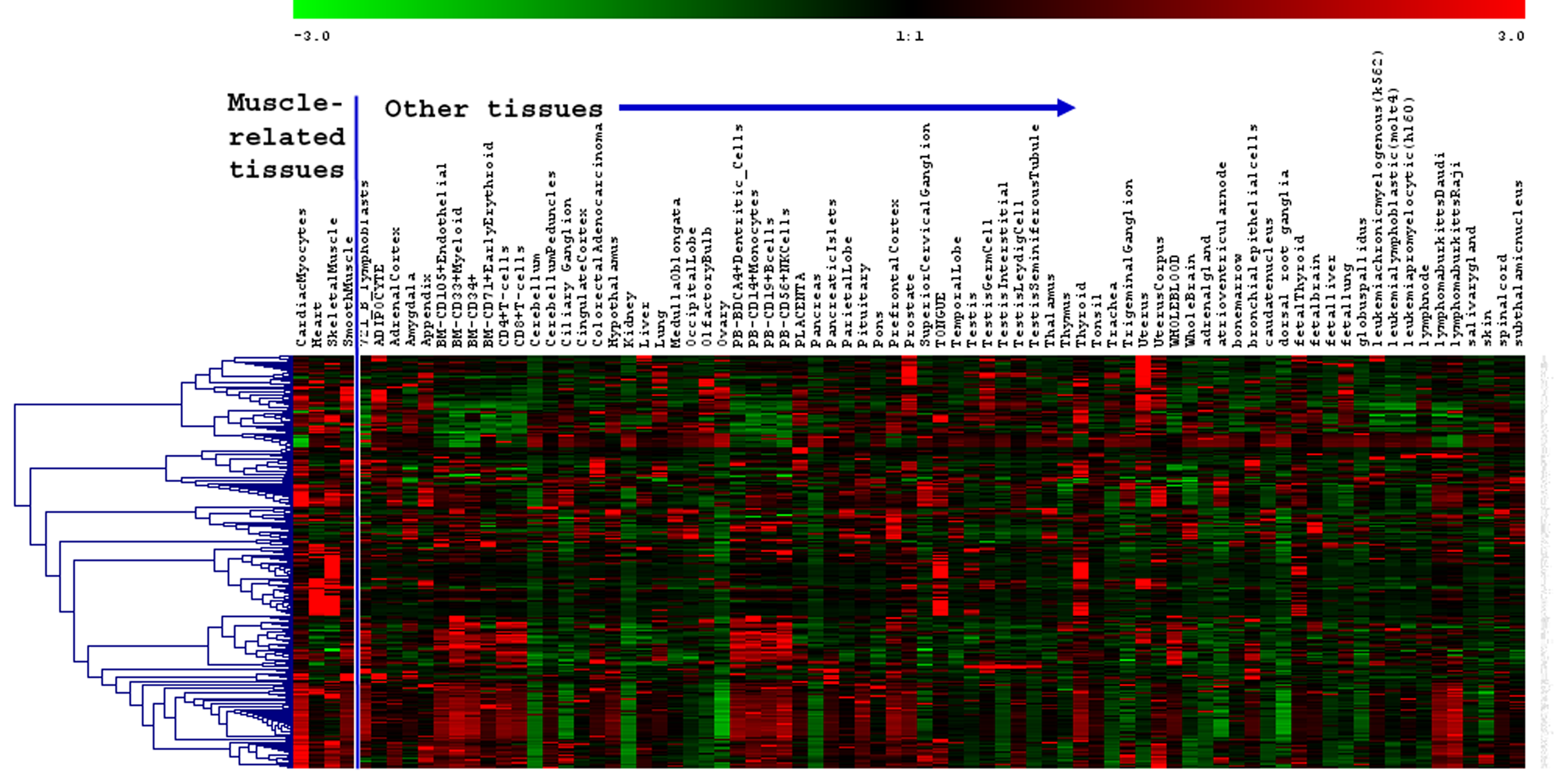

Supplement: Figure S3 — Sample-wise gene-level variations shown by the heatmap of Z-scores for sample-level differentiated genes, which are associated with the No. 1 (Muscle contraction) and No. 2 (Muscle development) terms from the SLEPR result in Table 9. The z-scores of these associated genes were computed using all samples from both Inclusion and Exclusion classes, and were displayed in the heatmap using color gradient for their values as red for positive z-scores and green for negative z-scores, black for scores of 0. (1.37 MB TIF) [file pone.0003288.s003.tif]
