# Supplementary material for: SLEPR: A Sample-Level Enrichment-Based Pathway Ranking Method — Seeking Biological Themes through Pathway-Level Consistency
Source: PLoS One. 2008 Sep 26;3(9):e3288. doi: 10.1371/journal.pone.0003288 (PMC2546449; doi:10.1371/journal.pone.0003288)
Supplement: Figure S4 — No bias for the SLEPR ranking scores vs. sizes of gene sets. A pdf file with several plots showing no bias for the SLEPR ranking scores vs. sizes of the gene sets: for distribution of SLEPR pathway ranking scores vs. gene set sizes (with different gene set size windows) and histogram distribution of gene set sizes for data in Table S2, Table S4, and Table S6 (for all terms or different numbers of top ranked terms in the tables). (1.92 MB PDF) [file pone.0003288.s004.pdf]

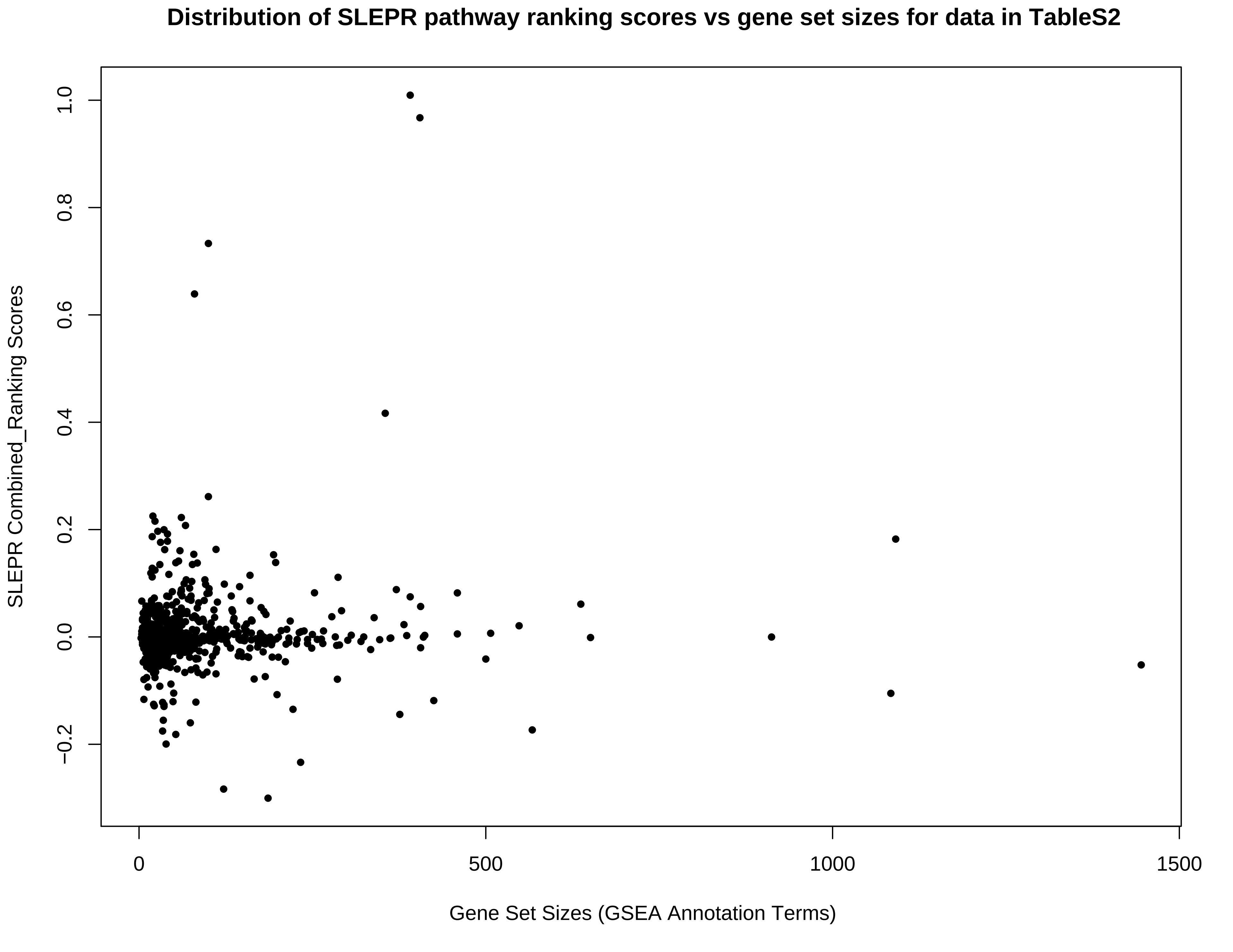
Distribution of SLEPR pathway ranking scores vs gene set sizes for data in TableS2
SLEPR Combined_Ranking Scores
1.0
0.8
0.6
0.4
0.2
0.0
−0.2
0
500
1000
1500
Gene Set Sizes (GSEA Annotation Terms)

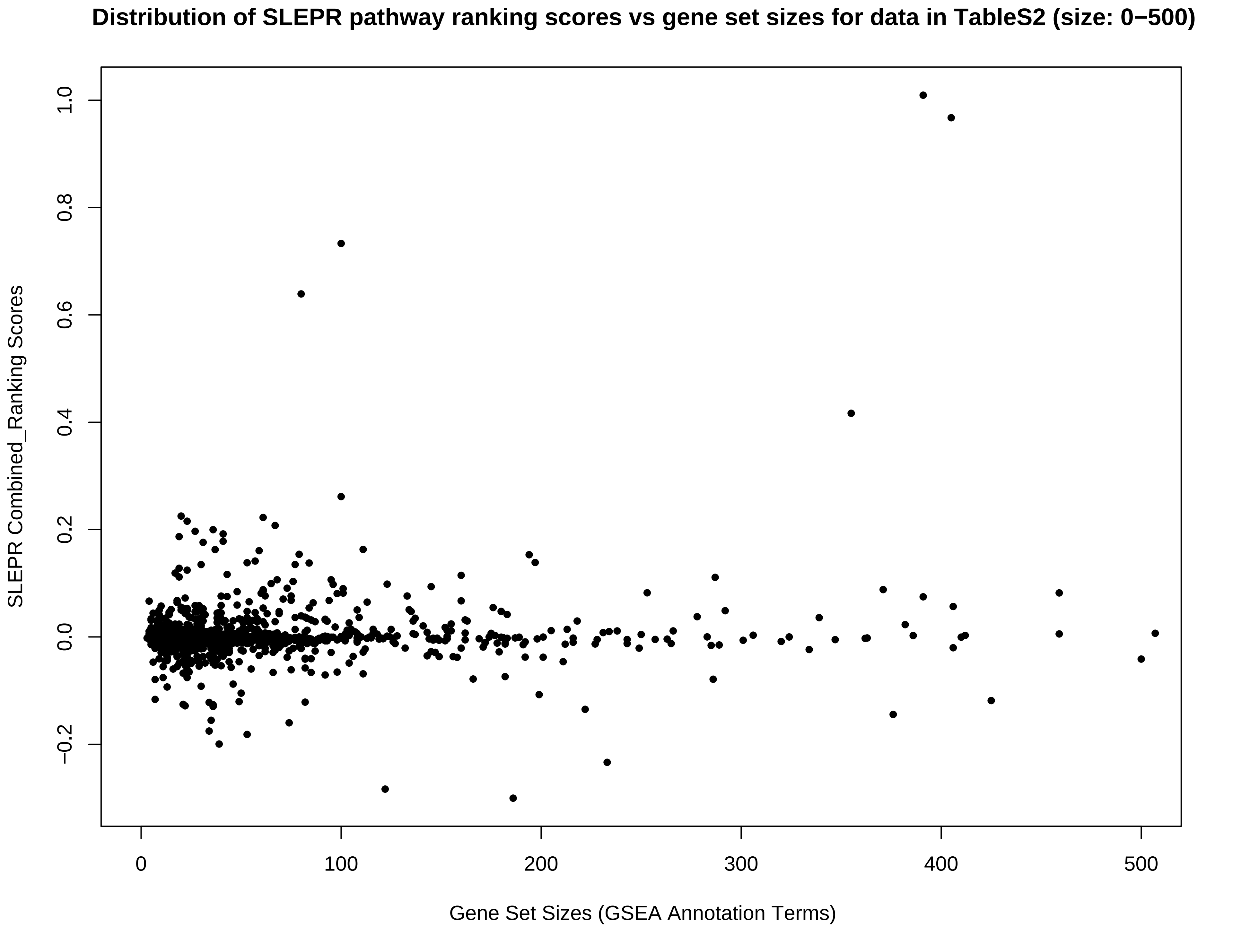
Distribution of SLEPR pathway ranking scores vs gene set sizes for data in TableS2 (size: 0−500)
SLEPR Combined_Ranking Scores
−0.2
0.0
0.2
0.4
0.6
0.8
1.0
0
100
200
300
400
500
Gene Set Sizes (GSEA Annotation Terms)

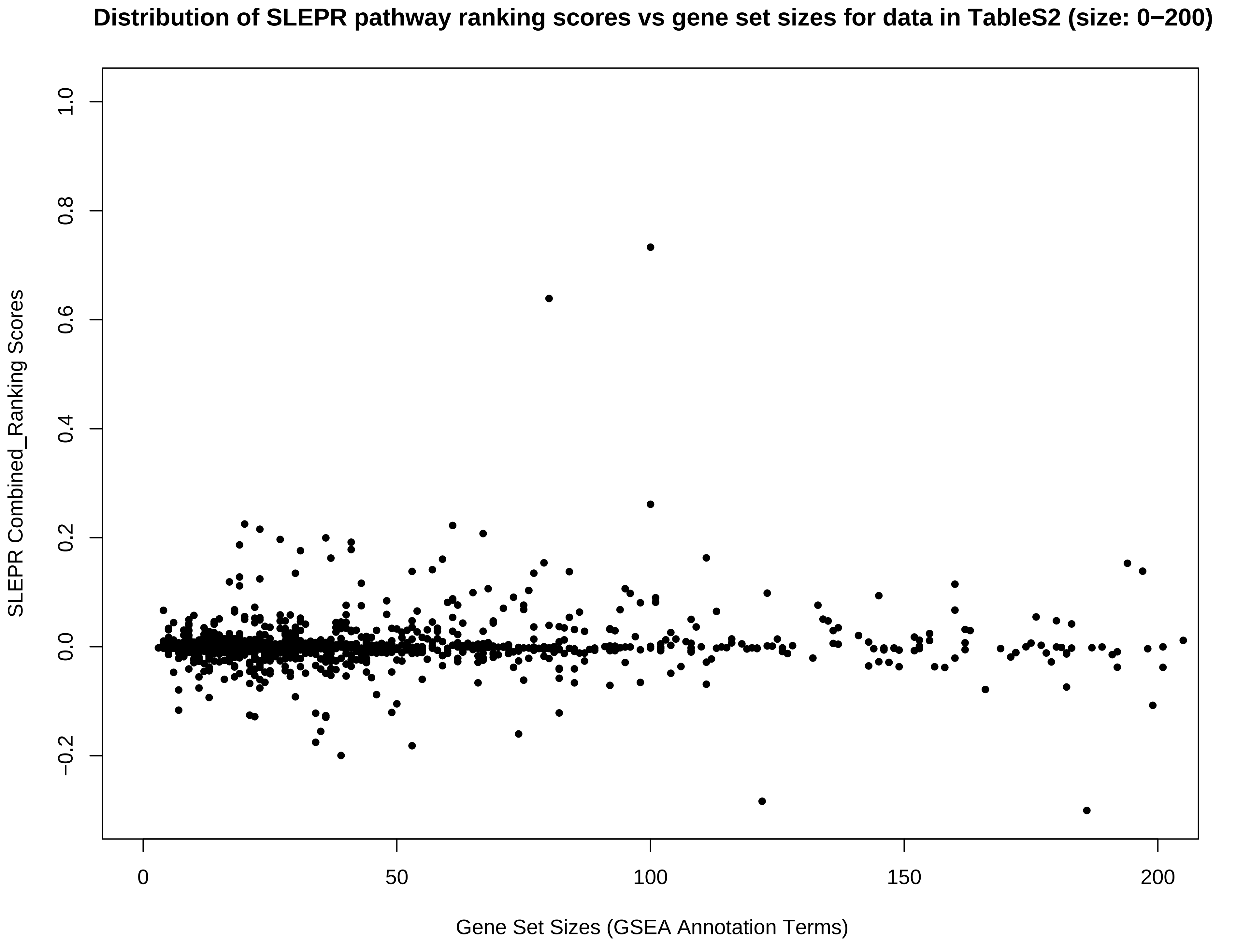
Distribution of SLEPR pathway ranking scores vs gene set sizes for data in TableS2 (size: 0−200)
SLEPR Combined_Ranking Scores
Gene Set Sizes (GSEA Annotation Terms)
−0.2
0.0
0.2
0.4
0.6
0.8
1.0
0
50
100
150
200

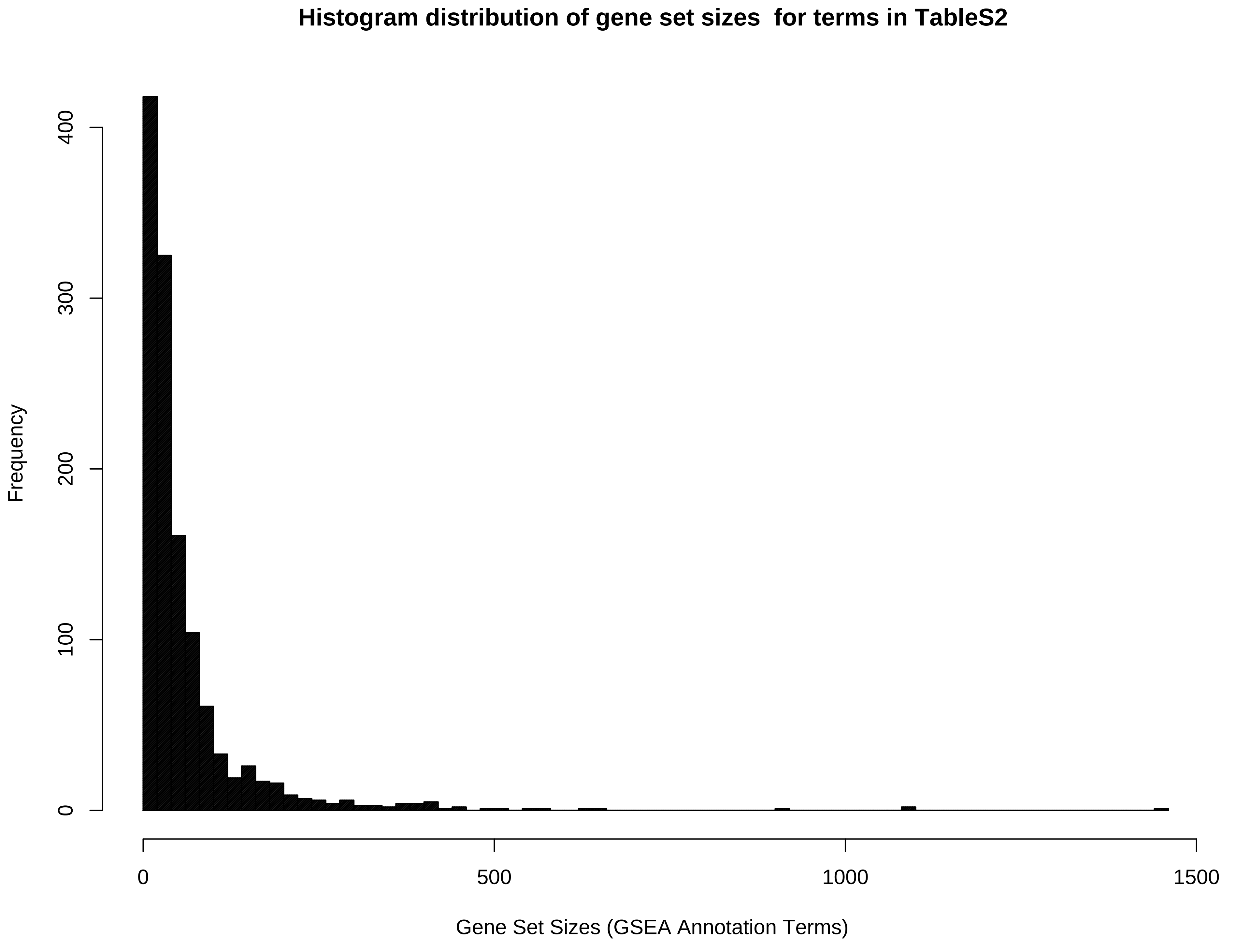
Histogram distribution of gene set sizes for terms in TableS2
Frequency
0
100
200
300
400
0
500
1000
1500
Gene Set Sizes (GSEA Annotation Terms)

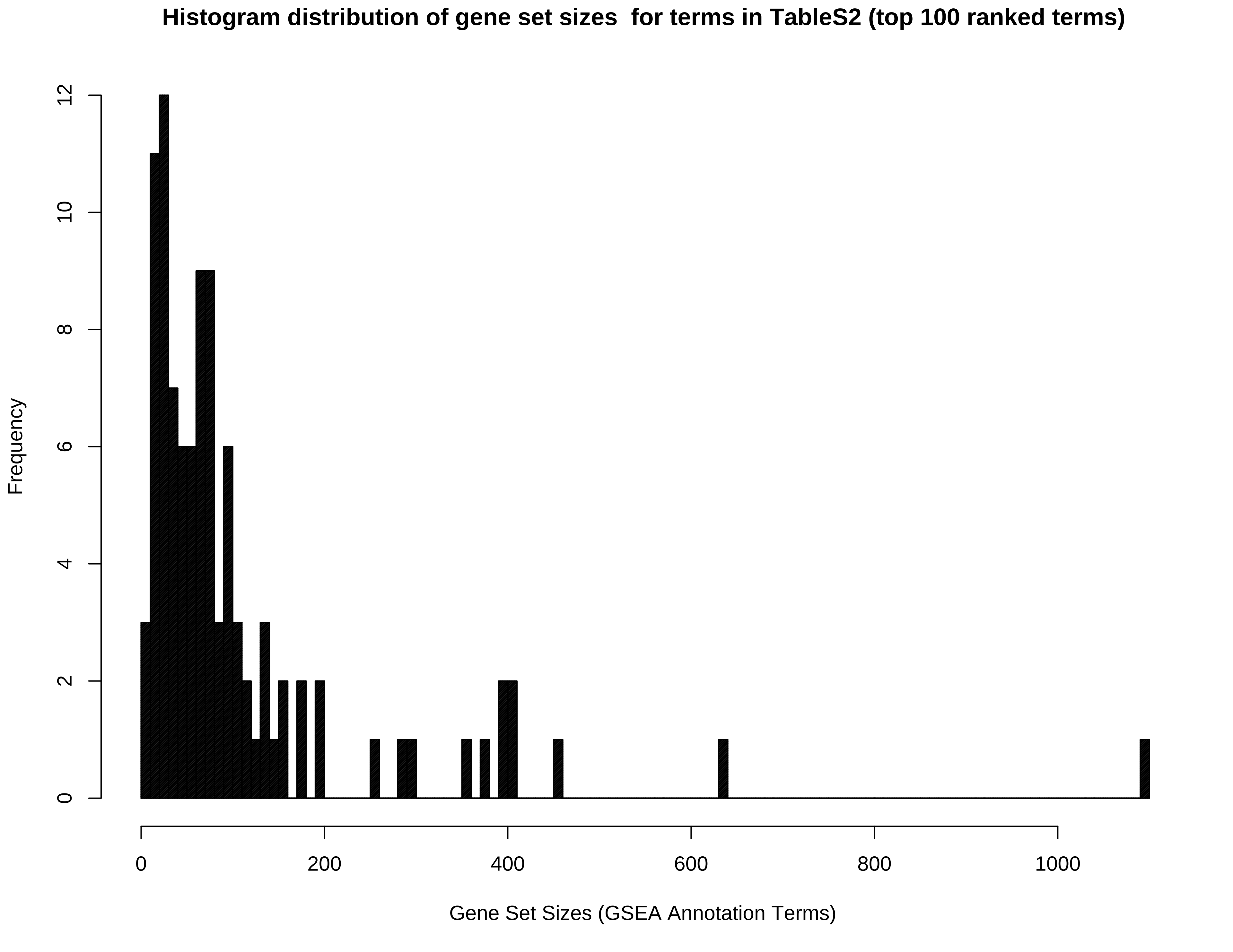
Histogram distribution of gene set sizes for terms in TableS2 (top 100 ranked terms)
Frequency
0
2
4
6
8
10
12
0
200
400
600
800
1000
Gene Set Sizes (GSEA Annotation Terms)

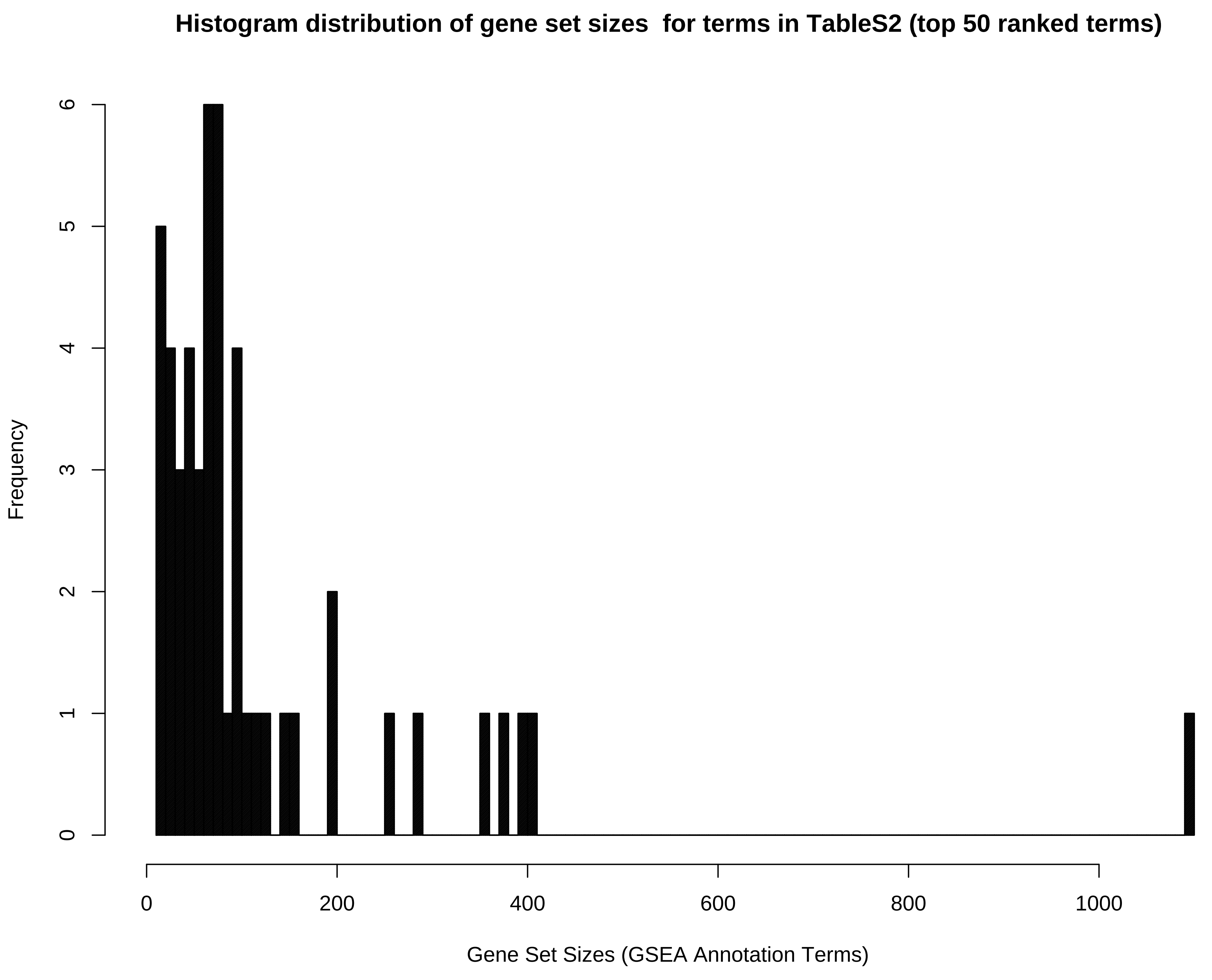

Histogram distribution of gene set sizes for terms in TableS2 (top 50 ranked terms)
Frequency
0
1
2
3
4
5
6
0
200
400
600
800
1000
Gene Set Sizes (GSEA Annotation Terms)

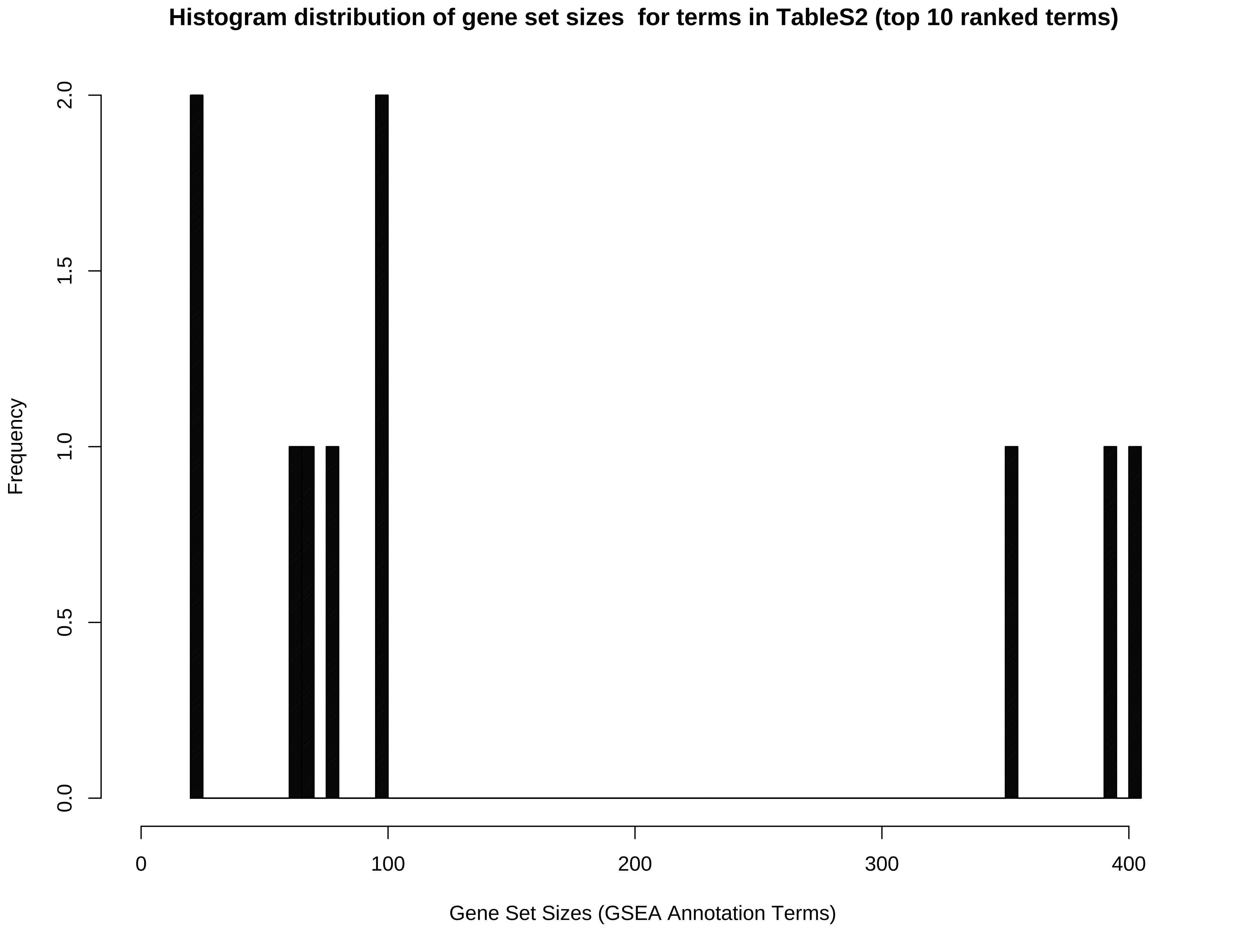
Histogram distribution of gene set sizes for terms in TableS2 (top 10 ranked terms)
Frequency
0.0
0.5
1.0
1.5
2.0
0
100
200
300
400
Gene Set Sizes (GSEA Annotation Terms)

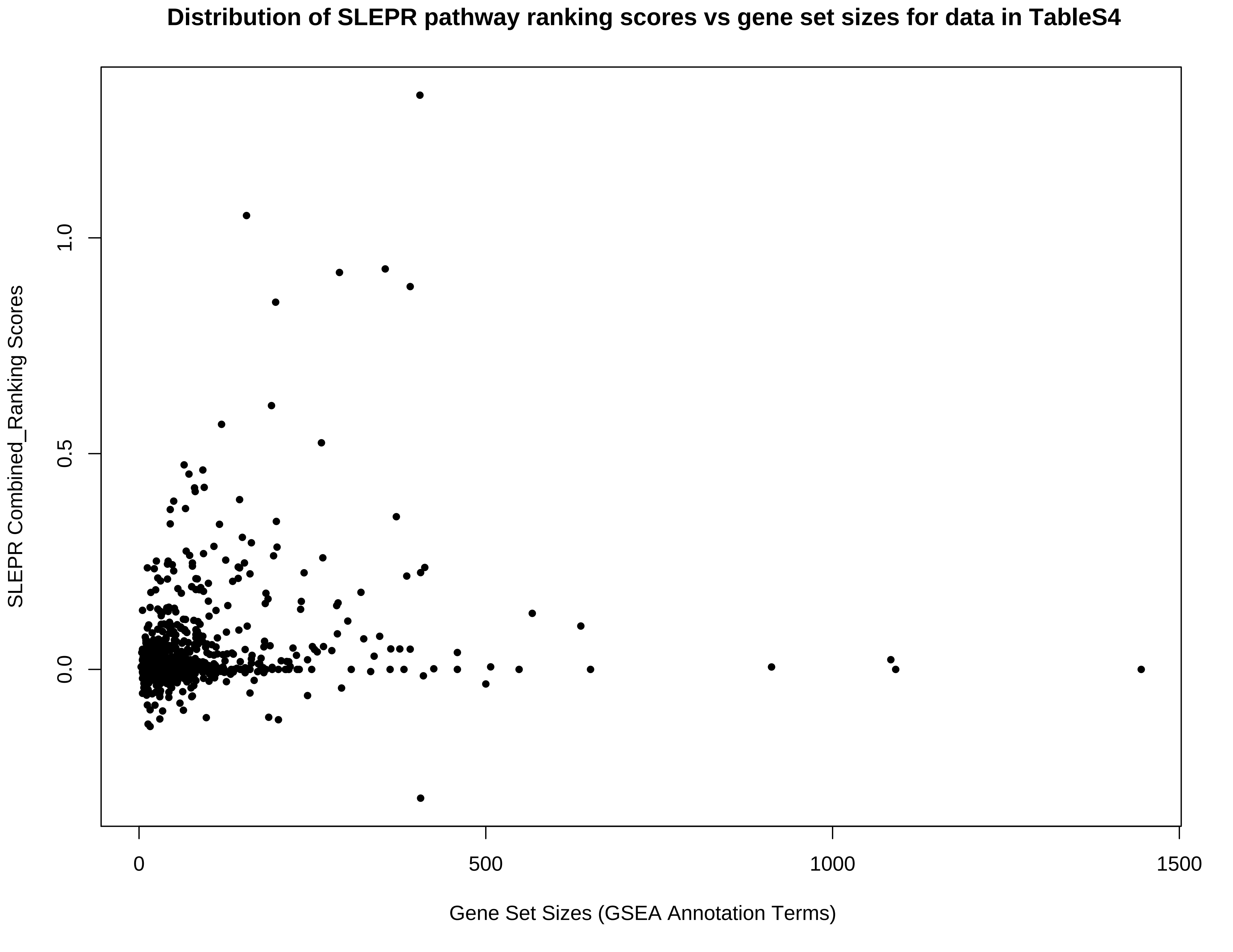
Distribution of SLEPR pathway ranking scores vs gene set sizes for data in TableS4
SLEPR Combined_Ranking Scores
0.0
0.5
1.0
0
500
1000
1500
Gene Set Sizes (GSEA Annotation Terms)

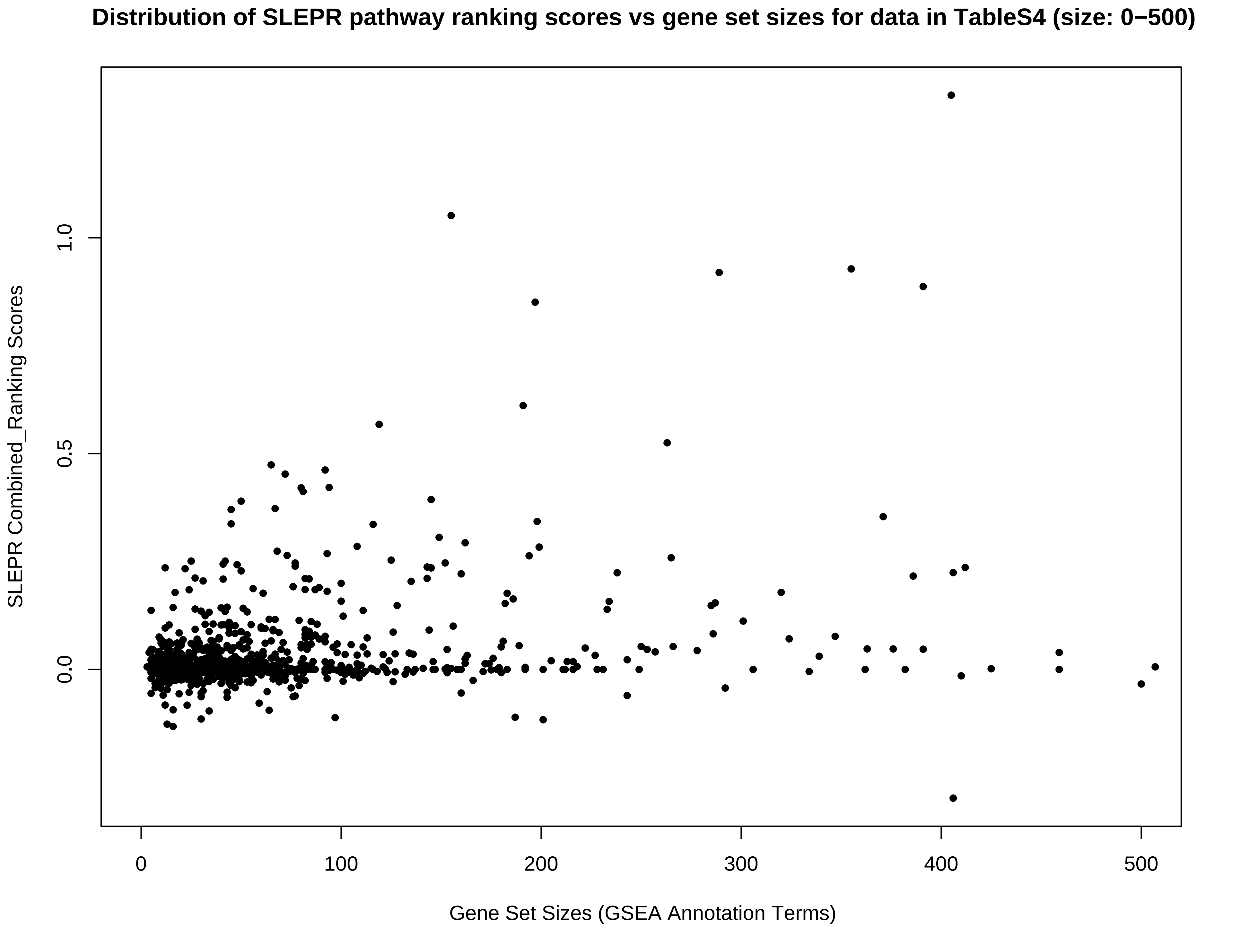

Distribution of SLEPR pathway ranking scores vs gene set sizes for data in TableS4 (size: 0−500)
SLEPR Combined_Ranking Scores
0.0
0.5
1.0
0
100
200
300
400
500
Gene Set Sizes (GSEA Annotation Terms)

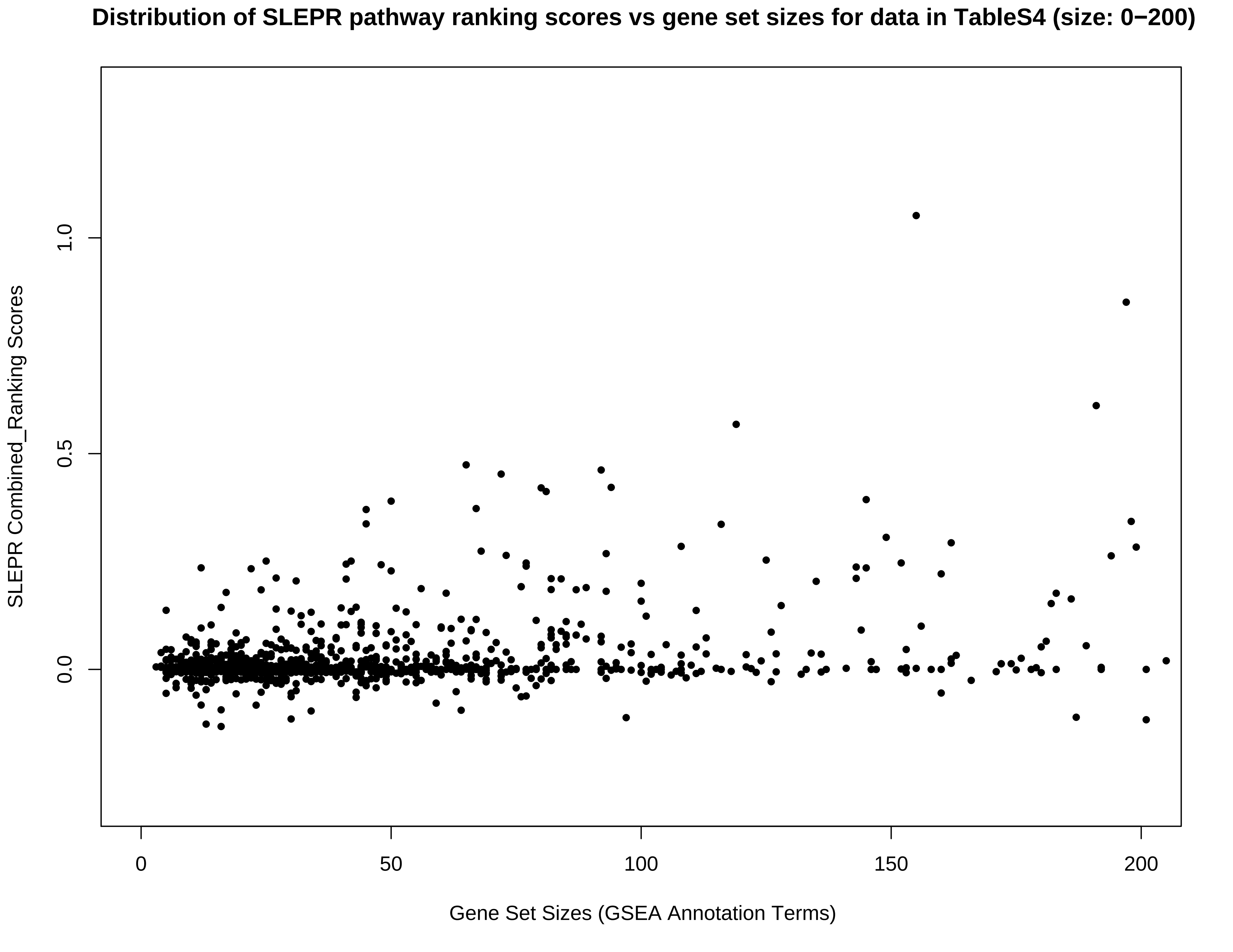
Distribution of SLEPR pathway ranking scores vs gene set sizes for data in TableS4 (size: 0−200)
SLEPR Combined_Ranking Scores
1.0
0.5
0.0
0
50
100
150
200
Gene Set Sizes (GSEA Annotation Terms)

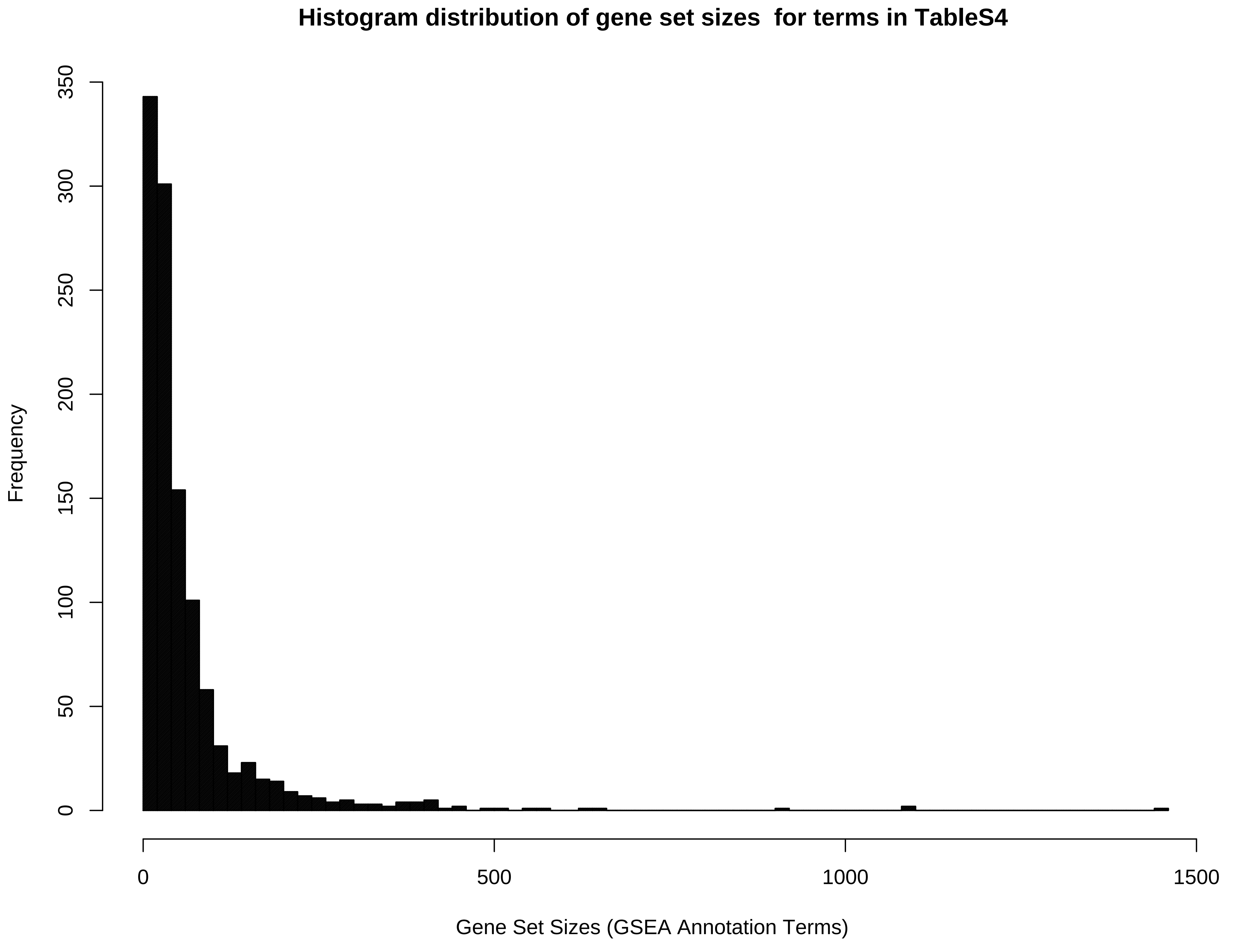
Histogram distribution of gene set sizes for terms in TableS4
Frequency
0
50
100
150
200
250
300
350
0
500
1000
1500
Gene Set Sizes (GSEA Annotation Terms)

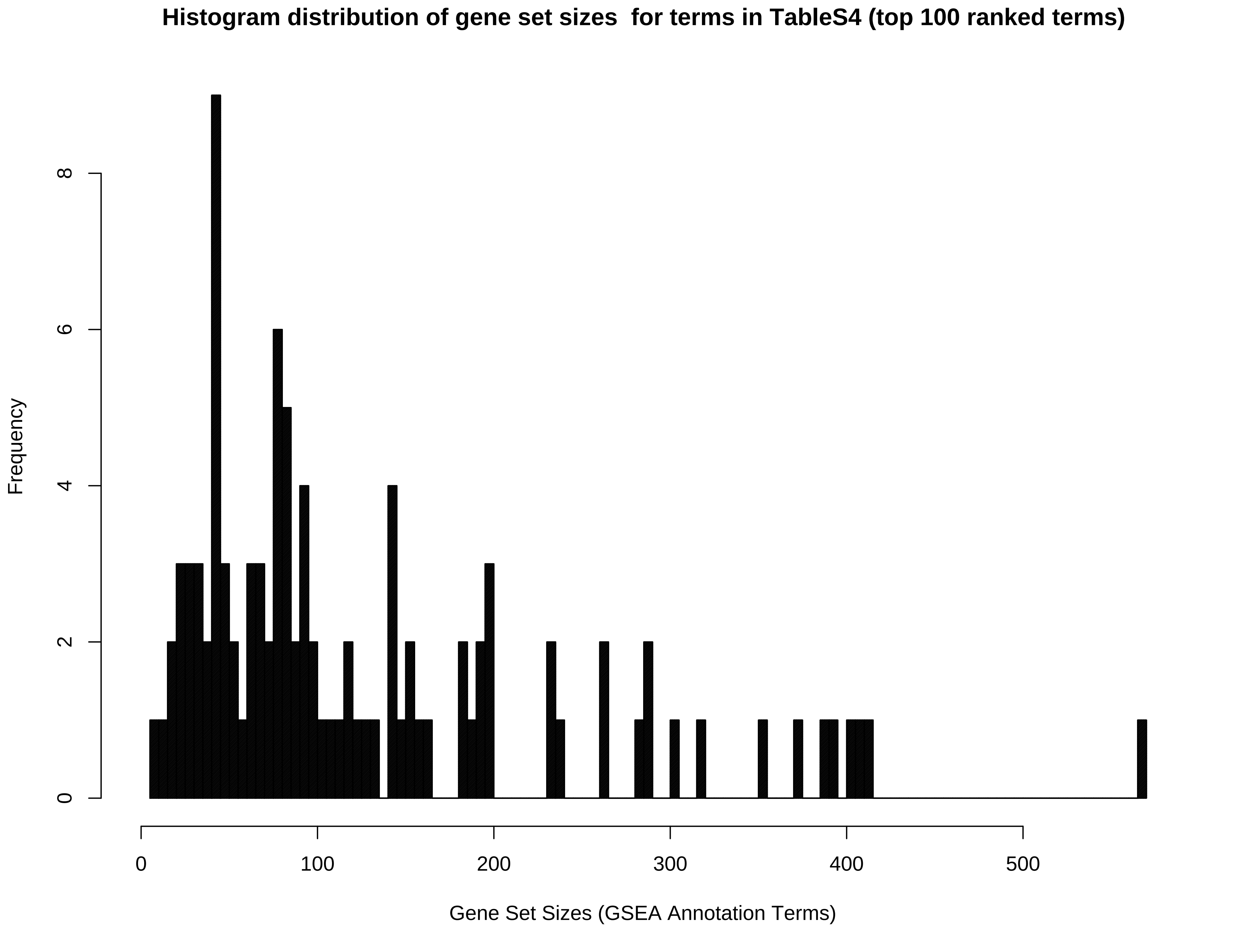
Histogram distribution of gene set sizes for terms in TableS4 (top 100 ranked terms)
Frequency
0
2
4
6
8
0
100
200
300
400
500
Gene Set Sizes (GSEA Annotation Terms)

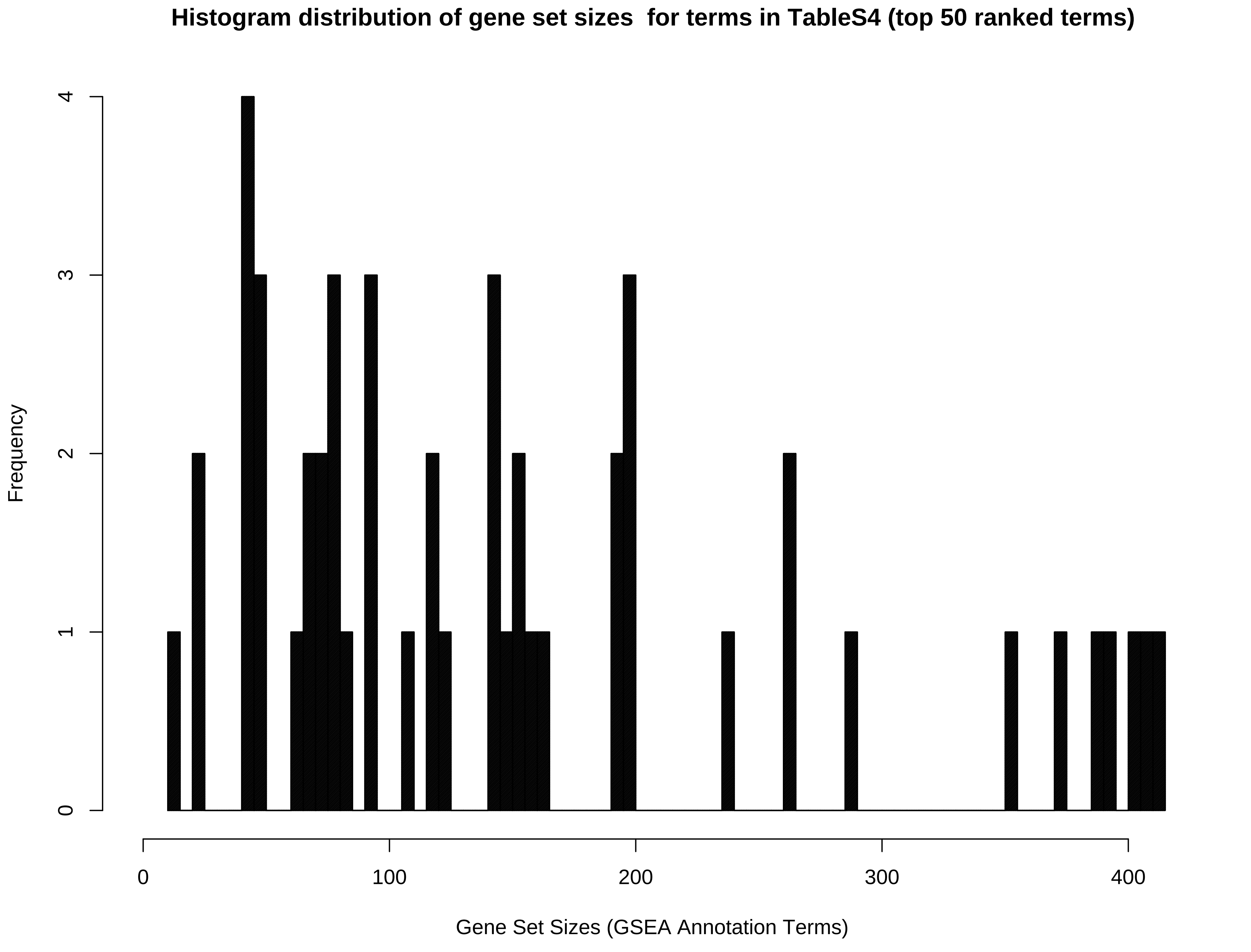
Histogram distribution of gene set sizes for terms in TableS4 (top 50 ranked terms)
Frequency
0
1
2
3
4
0
100
200
300
400
Gene Set Sizes (GSEA Annotation Terms)

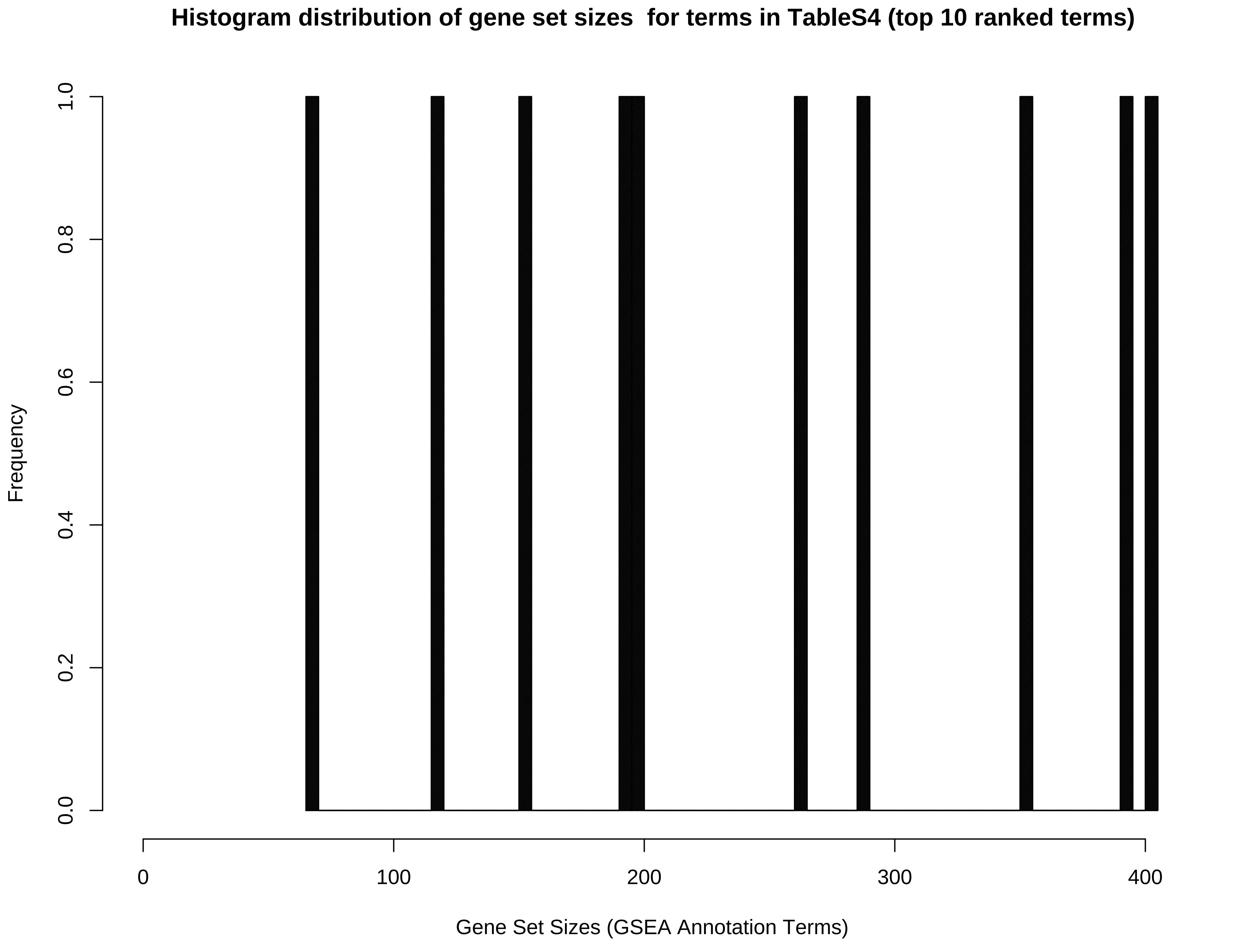
Histogram distribution of gene set sizes for terms in TableS4 (top 10 ranked terms)
Frequency
0.0
0.2
0.4
0.6
0.8
1.0
0
100
200
300
400
Gene Set Sizes (GSEA Annotation Terms)

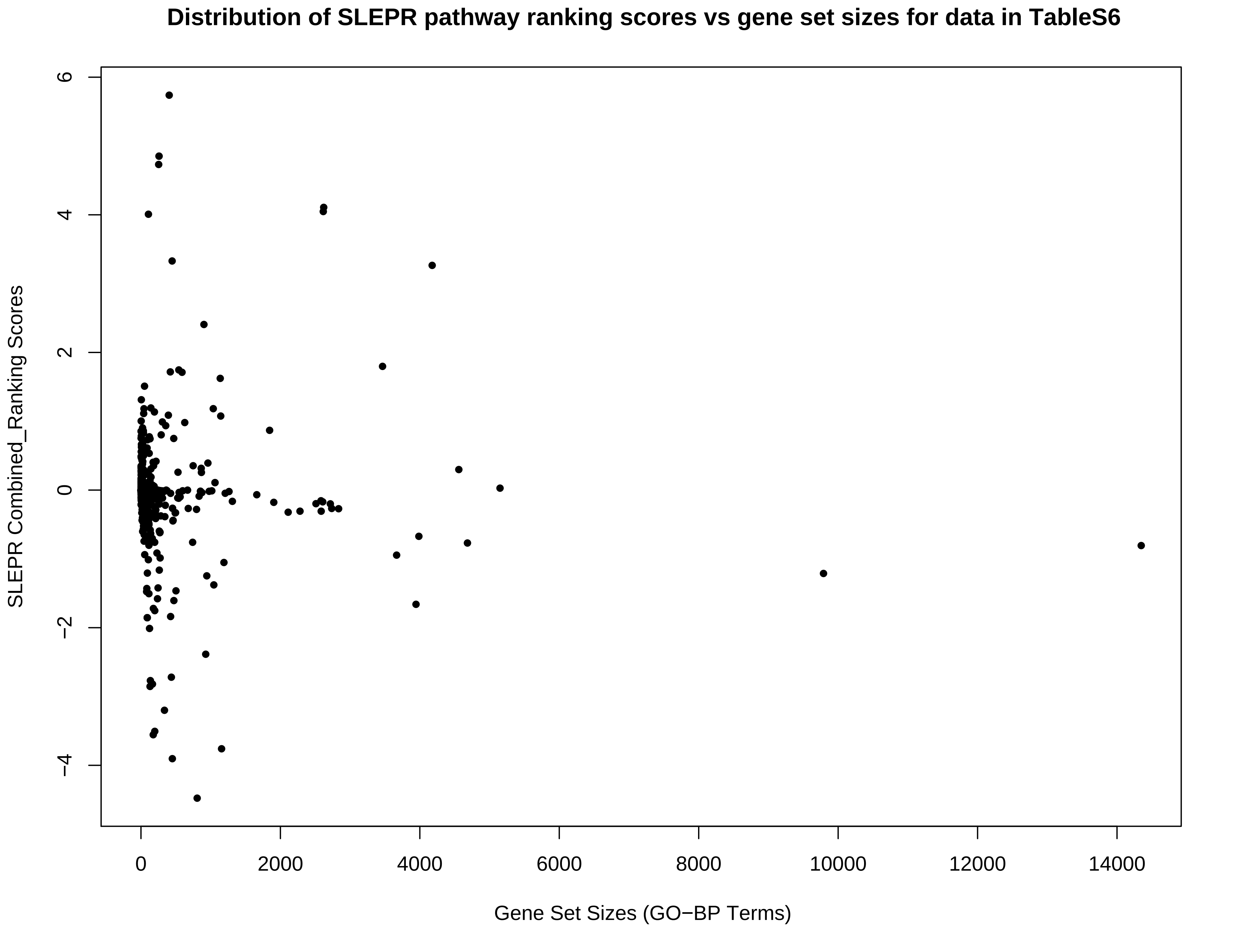
Distribution of SLEPR pathway ranking scores vs gene set sizes for data in TableS6
SLEPR Combined_Ranking Scores
−4
−2
0
2
4
6
0
2000
4000
6000
8000
10000
12000
14000
Gene Set Sizes (GO−BP Terms)

Distribution of SLEPR pathway ranking scores vs gene set sizes for data in TableS6 (size: 0−500)

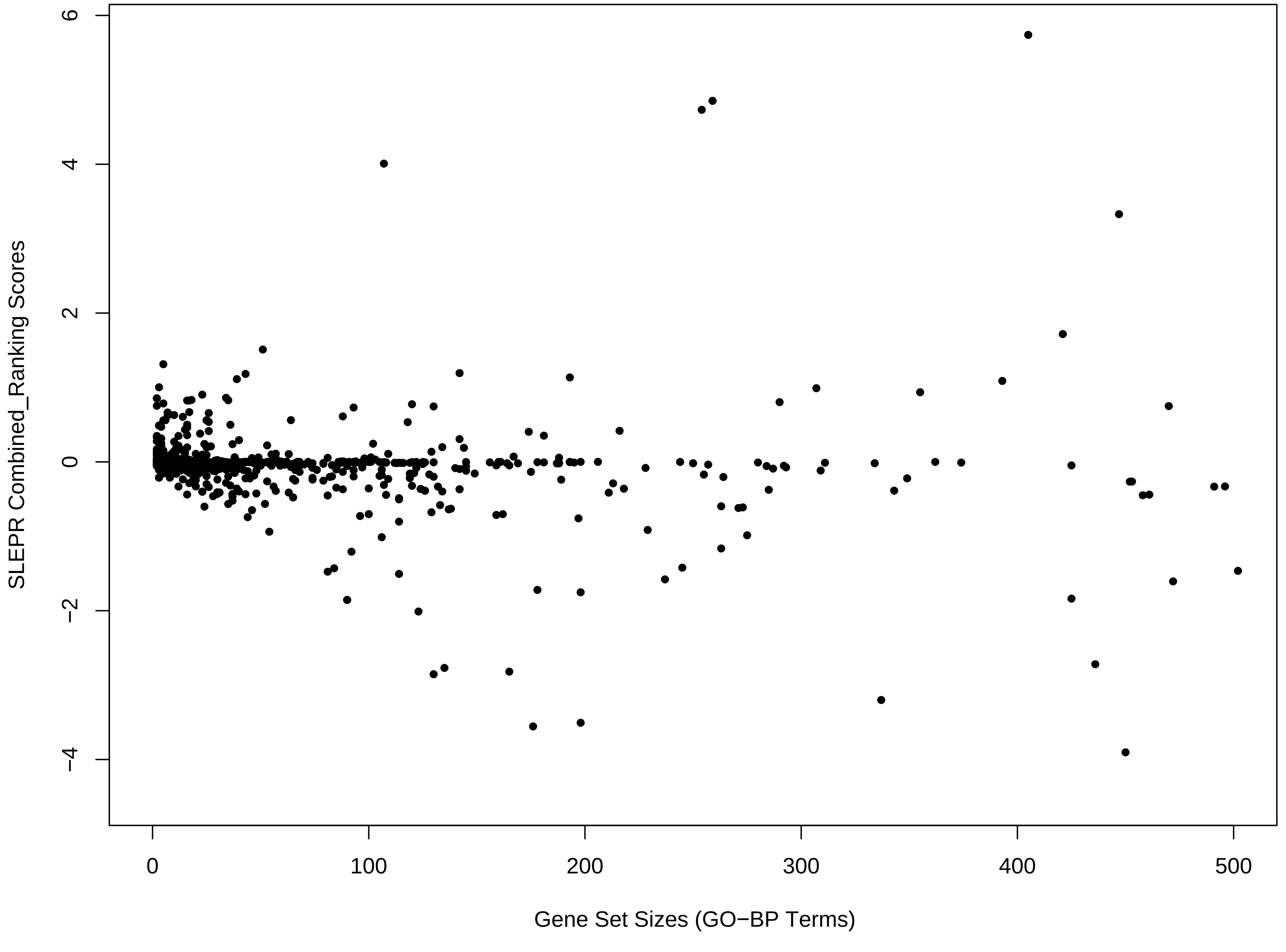

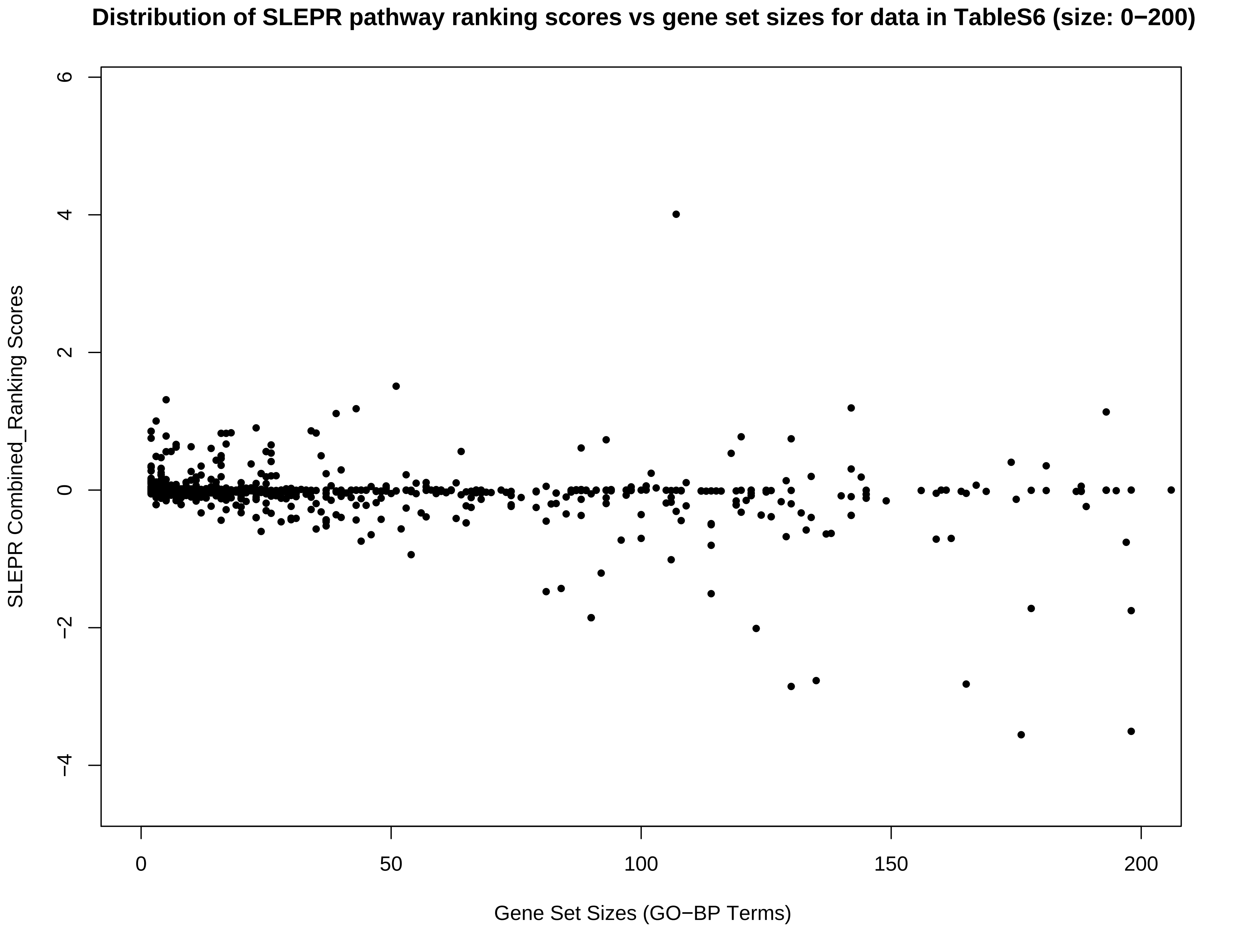
Distribution of SLEPR pathway ranking scores vs gene set sizes for data in TableS6 (size: 0−200)
SLEPR Combined_Ranking Scores
Gene Set Sizes (GO−BP Terms)
−4
−2
0
2
4
6
0
50
100
150
200

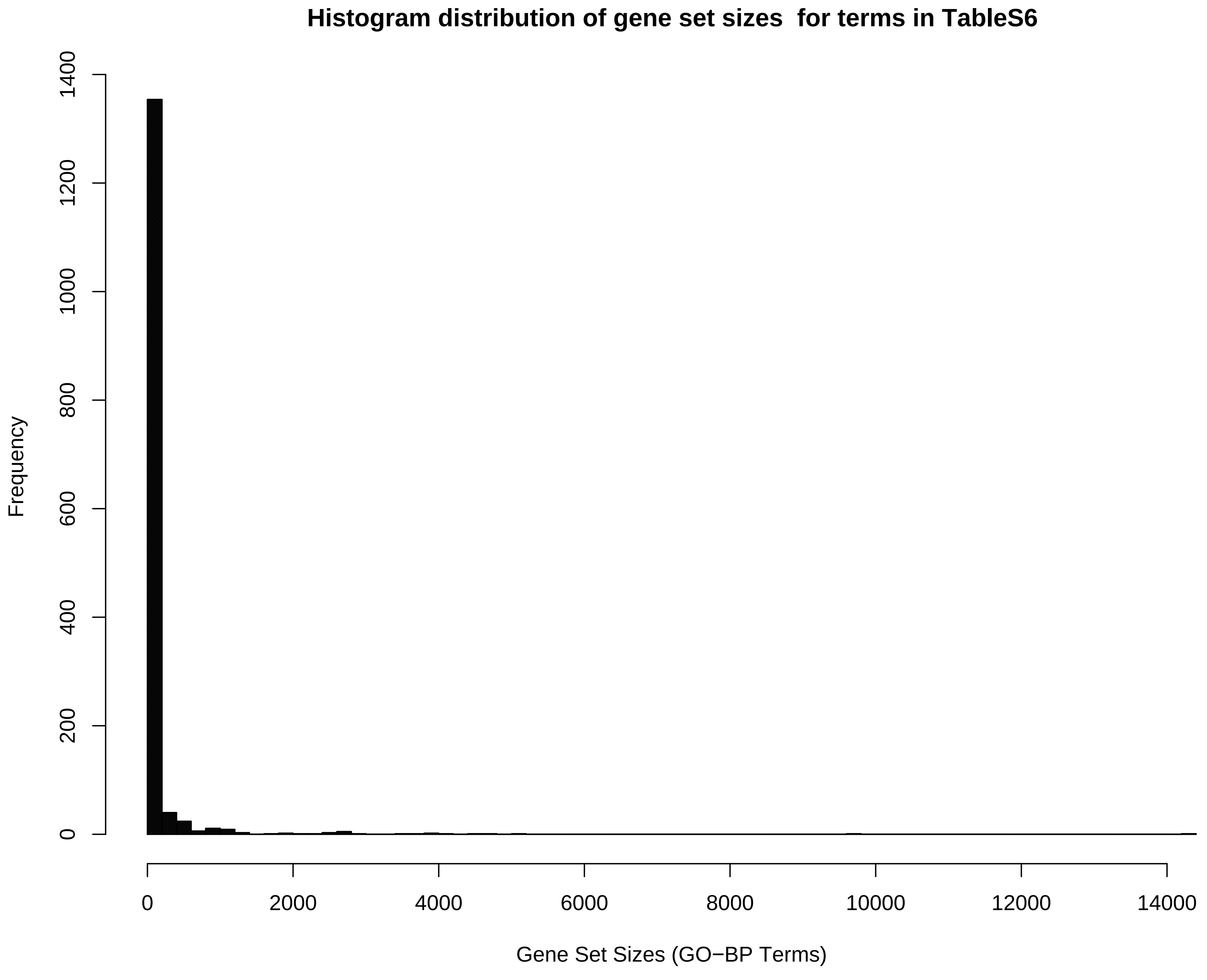
Histogram distribution of gene set sizes for terms in TableS6
Frequency
0
200
400
600
800
1000
1200
1400
0
2000
4000
6000
8000
10000
12000
14000
Gene Set Sizes (GO−BP Terms)

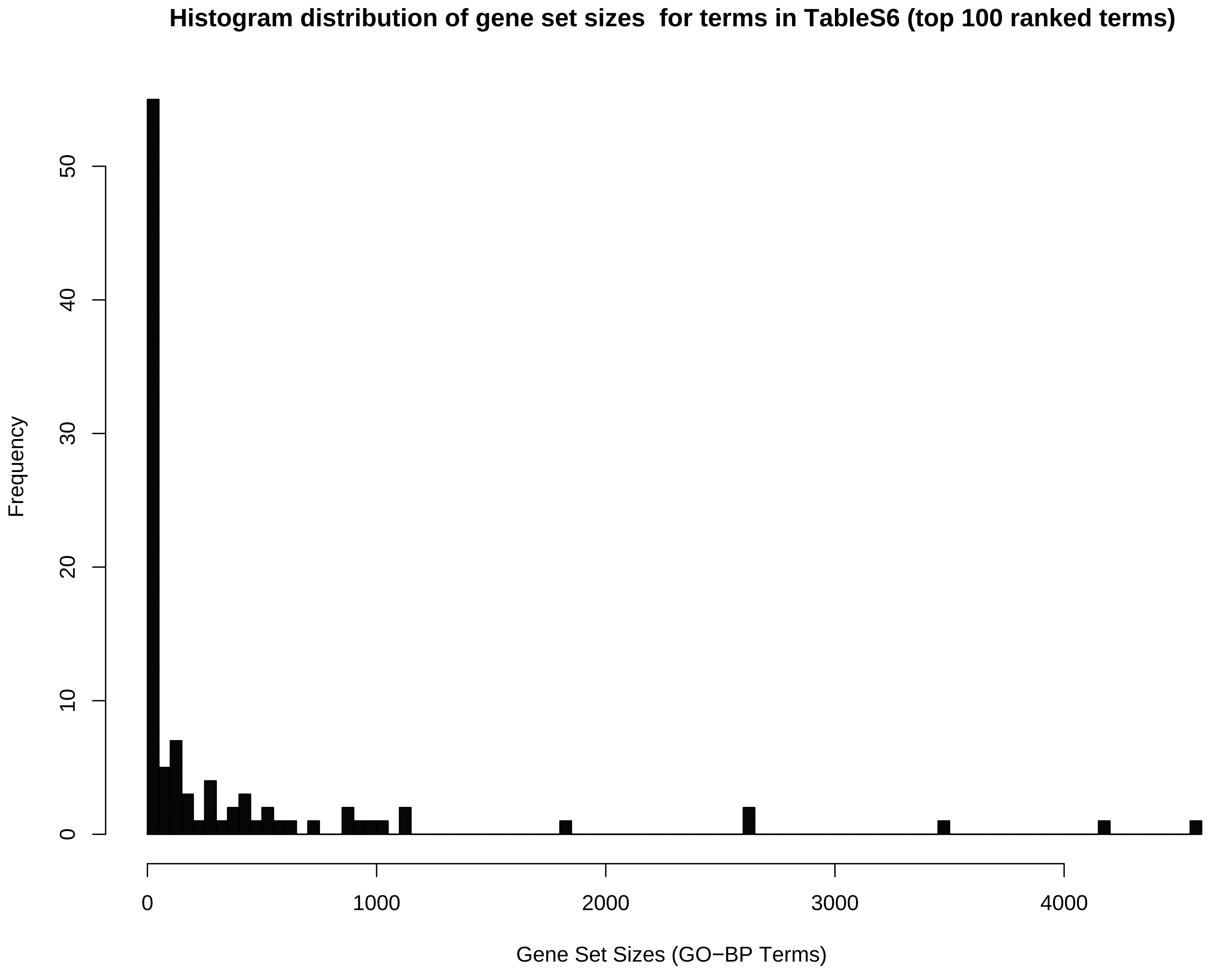
Histogram distribution of gene set sizes for terms in TableS6 (top 100 ranked terms)
Frequency
0
10
20
30
40
50
0
1000
2000
3000
4000
Gene Set Sizes (GO−BP Terms)

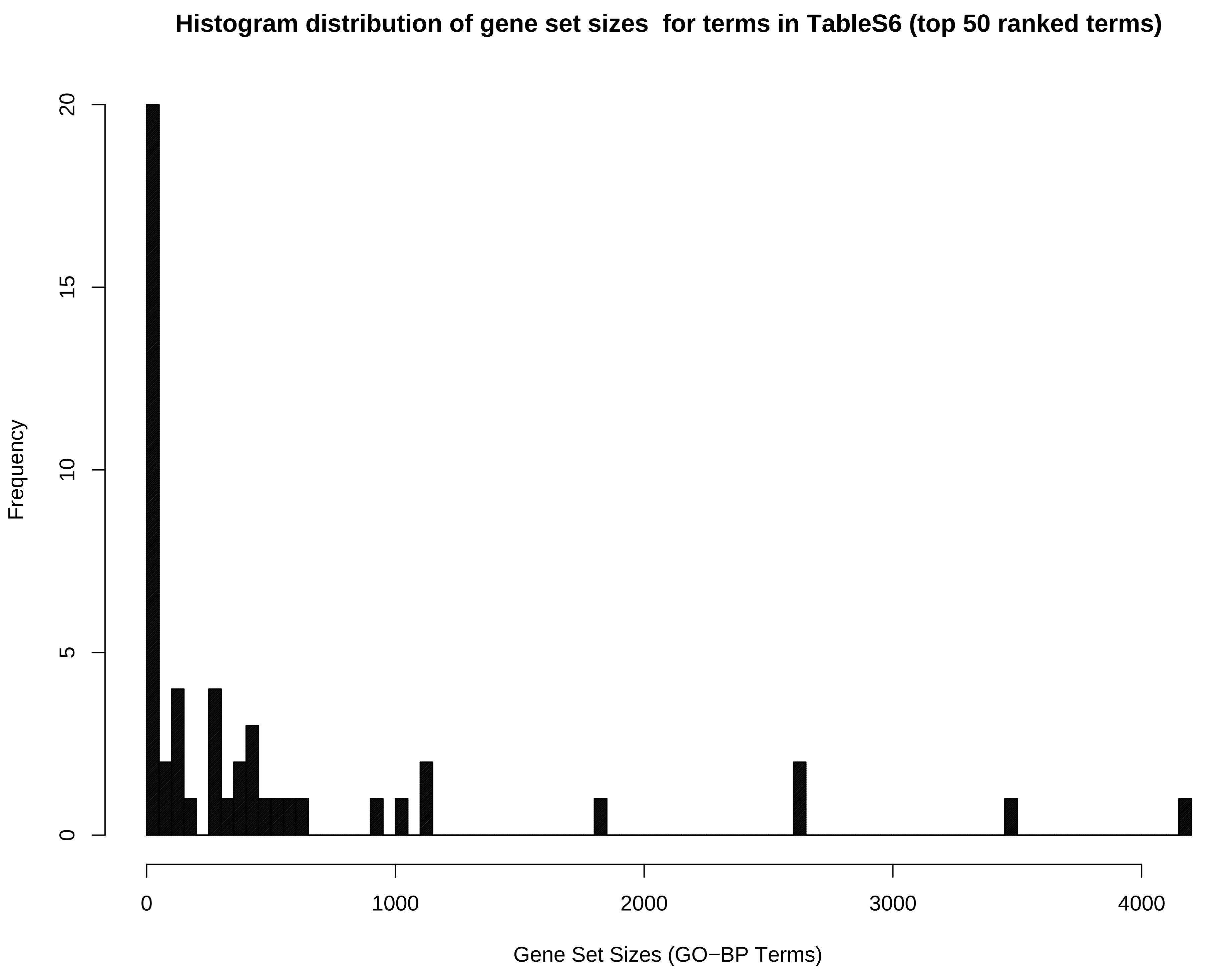
Histogram distribution of gene set sizes for terms in TableS6 (top 50 ranked terms)
Frequency
0
5
10
15
20
0
1000
2000
3000
4000
Gene Set Sizes (GO−BP Terms)

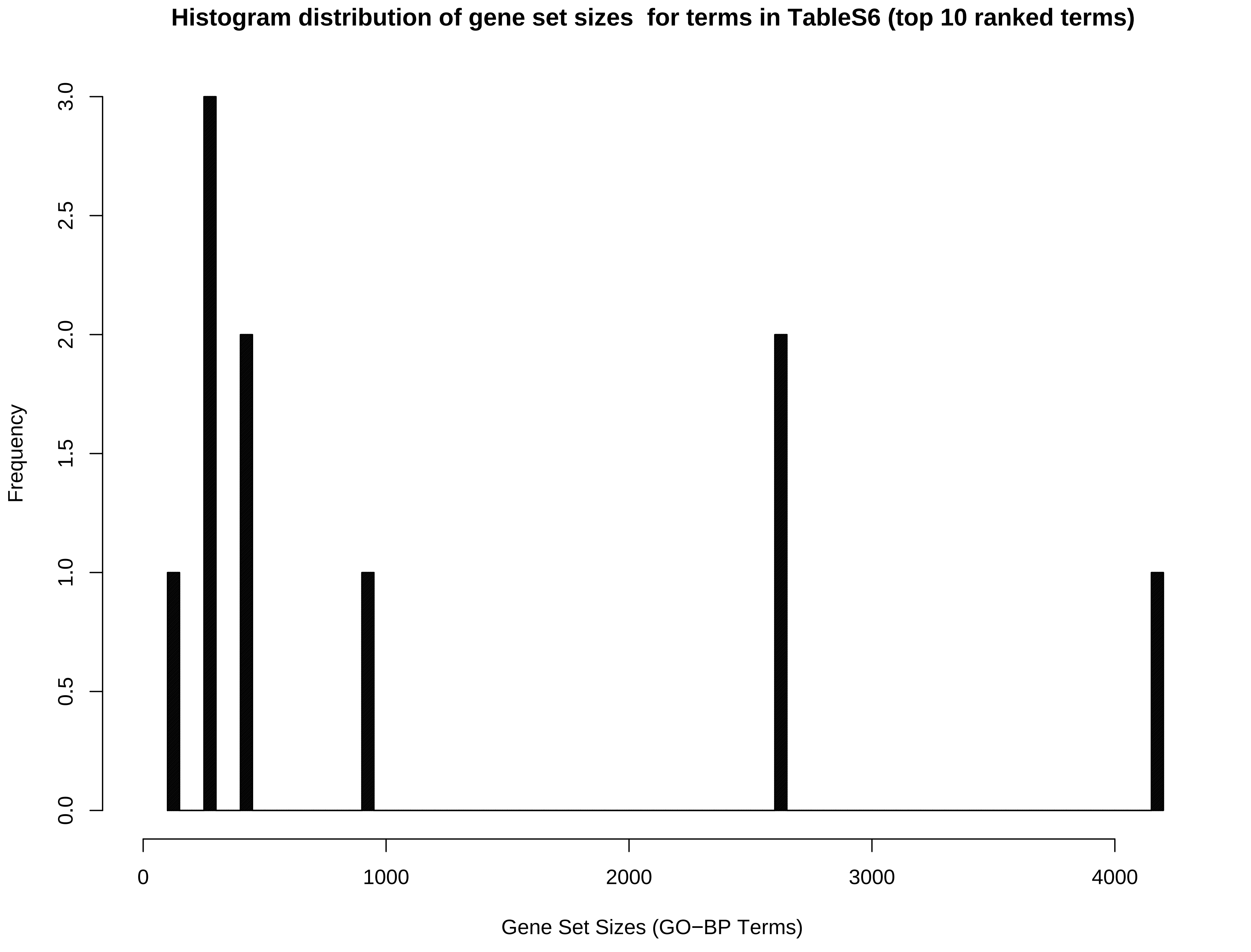
Histogram distribution of gene set sizes for terms in TableS6 (top 10 ranked terms)
Frequency
0.0
0.5
1.0
1.5
2.0
2.5
3.0
0
1000
2000
3000
4000
Gene Set Sizes (GO−BP Terms)
